# Atomistic design of microbial opsin-based blue-shifted optogenetics tools

**DOI:** 10.1038/ncomms8177

**Published:** 2015-05-15

**Authors:** Hideaki E. Kato, Motoshi Kamiya, Seiya Sugo, Jumpei Ito, Reiya Taniguchi, Ayaka Orito, Kunio Hirata, Ayumu Inutsuka, Akihiro Yamanaka, Andrés D. Maturana, Ryuichiro Ishitani, Yuki Sudo, Shigehiko Hayashi, Osamu Nureki

**Affiliations:** 1 Department of Biological Sciences, Graduate School of Science, The University of Tokyo, 2-11-16 Yayoi, Bunkyo-ku, Tokyo 113-0032, Japan; 2 Department of Chemistry, Graduate School of Science, Kyoto University, Kyoto 606-8502, Japan; 3 Department of Bioengineering Sciences, Graduate School of Bioagricultural Sciences, Nagoya University, Furo-cho, Chikusa-ku, Nagoya 464-8601, Japan; 4 RIKEN SPring-8 Center, Hyogo 679-5148, Japan; 5 Department of Neuroscience II, Research Institute of Environmental Medicine, Nagoya University, Nagoya 464-8601, Japan; 6 Division of Pharmaceutical Sciences, Graduate School of Medicine, Dentistry, and Pharmaceutical Sciences, Okayama University, 1-1-1 Tsushima-naka, Kita-ku, Okayama 700-8530, Japan

## Abstract

Microbial opsins with a bound chromophore function as photosensitive ion transporters and have been employed in optogenetics for the optical control of neuronal activity. Molecular engineering has been utilized to create colour variants for the functional augmentation of optogenetics tools, but was limited by the complexity of the protein–chromophore interactions. Here we report the development of blue-shifted colour variants by rational design at atomic resolution, achieved through accurate hybrid molecular simulations, electrophysiology and X-ray crystallography. The molecular simulation models and the crystal structure reveal the precisely designed conformational changes of the chromophore induced by combinatory mutations that shrink its π-conjugated system which, together with electrostatic tuning, produce large blue shifts of the absorption spectra by maximally 100 nm, while maintaining photosensitive ion transport activities. The design principle we elaborate is applicable to other microbial opsins, and clarifies the underlying molecular mechanism of the blue-shifted action spectra of microbial opsins recently isolated from natural sources.

## Abstract (web summary)

Retinal-bound opsins are widely used tools for optical control of neuronal activity *in vivo*, so called optogenetics. Here, using molecular simulations, biochemistry, electrophysiology and X-ray crystallography, the authors present new molecular design principles for the generation of blue-shifted variants of microbial rhodopsins.

## Graphical abstract

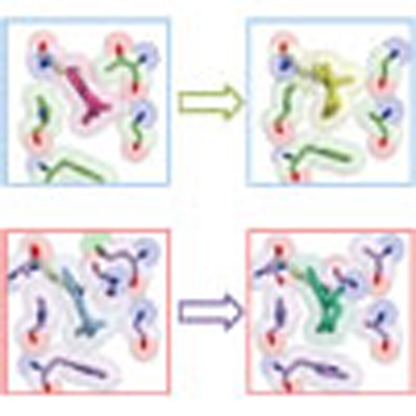

Photoreceptor proteins have been widely utilized as biotechnological tools in the genetic techniques to optically control cell activities, called optogenetics^1^,^2^. One of the major families of such protein tools consists of microbial opsins, membrane proteins characterized by seven transmembrane helices that bind a chromophore, retinal, to a lysine residue of the protein through a protonated Schiff base linkage, RPSB^3^. Rhodopsins, the opsin–retinal complexes, exist in a wide variety of microbial species and serve in various physiological functions, such as photosynthesis and phototaxis. In optogenetics, some microbial opsins such as channelrhodopsins (ChRs) and archaerhodopsin-3 (AR3), which function as light-sensitive ion transporters, are heterologously expressed in animal neurons to excite and silence them, and consequently to control animal behaviours by illumination with light^4^,^5^,^6^,^7^,^8^,^9^.

Similar to the fluorescent proteins utilized in cell visualization, the functionalities of the microbial opsins in optogenetics have been diversified through genomic searches for analogous light-sensitive ion transporters and molecular engineering^10^. Colour variants, which enable colour-regulated dual-light activations of ion transporters, exemplify such augmentations of the functionality^11^,^12^,^13^,^14^,^15^,^16^,^17^. As seen in the visual receptors and various microbial rhodopsins, and demonstrated in an extensively engineered retinal-binding protein^18^, the absorption maximum of the RPSB chromophore can be tuned in a wide range of the visible region through protein–chromophore interactions.

The strategy for developing the colour variants has involved extensive searches and screens, which in general, require vast resources and would quickly reach a limit for further extension due to combinatorial explosion. Alternatively, a rational approach of molecular engineering based on solid design principles could circumvent this problem. Nevertheless, a rational approach to create colour variants of microbial rhodopsins with large spectral shifts has remained challenging, even though their three-dimensional structures are available. For example, point mutations at 13 positions introduced in phoborhodopsin (*λ*_max_=497 nm), guided based on the X-ray crystallographic structure^19^,^20^,^21^,^22^ to make the sequential and spatial alignments of the mutated residues in the binding pocket identical to those of a largely red-shifted microbial opsin, bacteriorhodopsin from *Halobacterium salinarum* (HsBR, *λ*_max_=568 nm), a light-driven proton pump, shifted the absorption maximum by 30 nm, which is only 40% of the overall 70 nm spectral shift of the native proteins^23^. This observation indicated that the creation of colour variants requires precise structural design at atomic resolution based on physicochemical considerations.

In the present study, we rationally develop largely blue-shifted colour variants of microbial rhodopsins, using theoretical chemistry, biochemistry, electrophysiology and X-ray crystallography. We formulate atomistic design principles of synergetic point mutations that induce conformational changes of the chromophore, consequently leading to a large spectral shift. Together with a point mutation that alters the electrostatic environment, we successfully obtain—with only double to quadruple point mutations—colour variants of a number of microbial rhodopsins exhibiting remarkable spectral shifts of up to 100 nm. This is twice as large as the maximum shift previously obtained by molecular engineering, while maintaining the photosensitive ion transport activities. The present study demonstrates that highly resolved atomistic design provides a strong basis for the production of colour variants through minimal modification that is universally applicable to analogous proteins.

## Results

### Atomistic design principle

**Figure 1:**
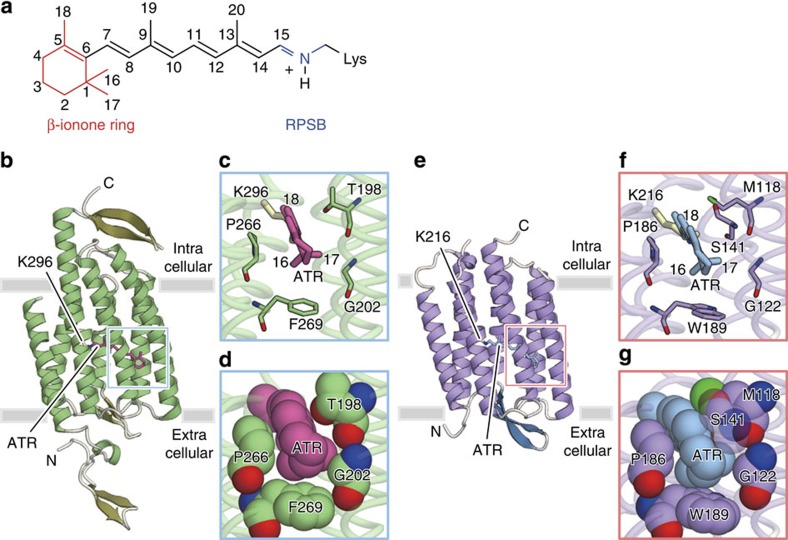
Opsin and chromophore structures of C1C2 and HsBR. (**a**) Chemical structure of the chromophore, RPSB, and its atom numbering. (**b**–**d**) Protein structure of C1C2 (PDB 3UG9). Overall structure (**b**), and close-up views of the chromophore binding pocket around the β-ionone ring of the all-*trans* retinal (ATR) in licorice (**c**) and van der Waals (vdW) (**d**) representations. (**e**–**g**) Protein structure of HsBR (PDB ID: 1C3W). Overall structure (**e**), and close-up views of the chromophore binding pocket around the β-ionone ring of ATR in licorice (**f**) and vdW (**g**) representations.

We first designed colour variants of two of the most widely used optogenetics tools, ChR and AR3, based on the available crystal structures of C1C2 (ref. 24), a chimeric protein of ChRs that has been utilized in optogenetics^25^,^26^,^27^, and HsBR, as HsBR shares high sequence similarity with AR3. In the native proteins of C1C2 and AR3, as well as those of microbial rhodopsins with presently available X-ray crystallographic structures, the methyl groups at positions 1 and 5 (C_16_, C_17_, and C_18_ methyl groups; see Fig. 1a) in the β-ionone ring of the chromophore are recognized by cavities created by the well-conserved residues of the binding pocket, which maintain the orientation of the ring alignment in the same plane as the polyene chain (Fig. 1b–g, Supplementary Fig 1 and Supplementary Table 1). Accordingly, the planar conformation at the C_6_–C_7_ bond, which connects the β-ionone ring with the polyene chain part, is fixed.

**Figure 2:**
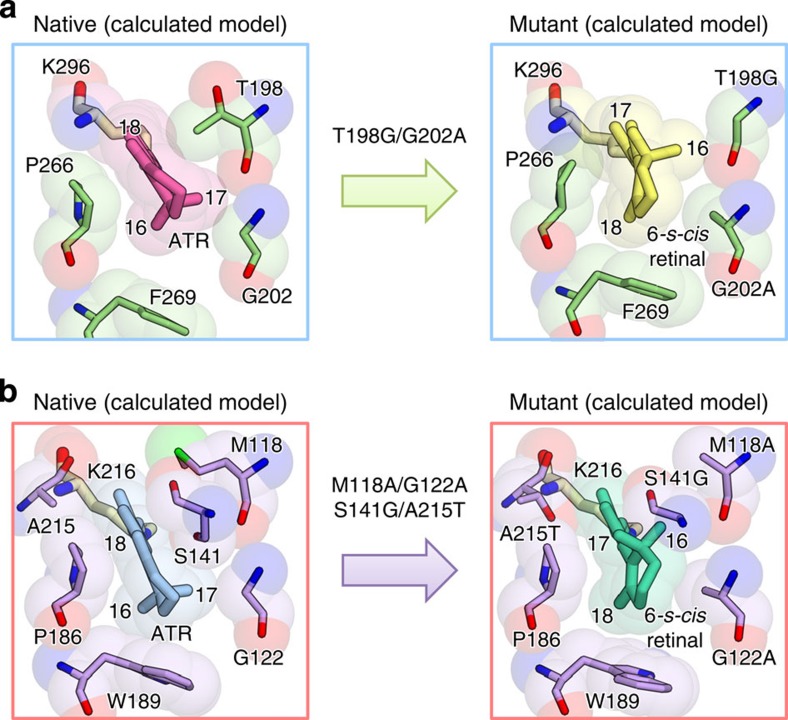
Structural models of blue-shifted mutants. Structural models of blue-shifted mutants of C1C2 (**a**) and HsBR (**b**), determined by QM/MM RWFE-SCF calculations.

We introduced point mutations that enforce torsion around the C_6_–C_7_ bond from planarity in C1C2 and AR3 (Fig. 2), to shrink the π-conjugation and consequently to produce blue shifts of their absorption spectra^3^,^28^ (Supplementary Fig. 2). The C_6_–C_7_ bond can be easily twisted, that is, the potential energy curve along the torsional coordinate is flat^29^,^30^,^31^ (Supplementary Fig. 2). Theoretical studies^30^,^31^,^32^,^33^,^34^,^35^ have suggested that the significant torsion around the C_6_–C_7_ bond by ∼30° in bovine rhodopsin (Supplementary Fig. 1 and Supplementary Table 1) is an important factor determining its absorption spectrum^36^.

The mutation-enforced torsion around the C_6_–C_7_ bond was designed as follows (see also Fig. 2). First, the β-ionone ring was rotated by ∼140°, thus forming the 6*-s-cis* conformation. In this rotated conformation, the C_18_ methyl group, instead of the C_16_ methyl group before the rotation, plunges into the cavity between Pro266 and Phe269 in C1C2. The rotation also moves the C_17_ methyl group to the cavity where the C_18_ methyl group was located before the rotation. These two methyl groups therefore avoid steric conflict in the binding pocket, by the rotation of the β-ionone ring. On the other hand, the rotation induces the steric overlap of the C_16_ methyl group and the moiety at position 2 in the β-ionone ring with the side chains of Thr198 in C1C2. To remove the steric overlap, we replaced the amino acid with glycine, which has a smaller side chain. In the case of HsBR, Met118 and Ser141, which are located in the region corresponding to Thr198 in C1C2, are replaced with alanine and glycine. In addition, the rotation creates a cavity at Gly202 in C1C2 (Gly122 in HsBR), which is filled by the C_17_ methyl group in the β-ionone ring before the rotation. Therefore, we replaced the glycine with alanine (and valine for AR3) to fill the created cavity. Overall, the double and triple mutations of the amino acids around the β-ionone ring described above were introduced to stabilize its rotated conformation in the binding pocket. Further shifts due to electrostatic modulation were also introduced into AR3 by the rotation of the β-ionone ring and an additional mutation, A225T. Generally speaking, a more negative (positive) field around the protonated Schiff base (the β-ionone ring) side of the chromophore shifts the absorption maximum to a shorter wavelength^3^,^28^. In the case of AR3, therefore, Ala225 (Ala215 in HsbR depicted in Fig. 2b) in the vicinity of the protonated Schiff base was replaced by threonine, to induce a further blue shift^37^,^38^,^39^,^40^. Moreover, the π-conjugation end of retinal and the neighbouring hydroxyl group of S151 (S141 in HsBR) in native AR3 are separated by the rotation of the β-ionone ring (Fig. 2b), giving rise to an additional blue-shift by weakening the electrostatic interaction.

Structural models of C1C2 (Fig. 2a) and HsBR (Fig. 2b) were obtained by free energy geometry optimizations with the QM/MM RWFE-SCF method^41^,^42^ (Fig. 2 and Supplementary Table 2). Free energetically stable structures with strongly twisted conformations of the C_6_–C_7_ bond were determined for the designed mutants, T198G/G202A of C1C2 and M118A/G122A/S141G/A215T of HsBR (Fig. 2 and Supplementary Table 2). The C_6_–C_7_ bond in the C1C2 mutant exhibited significant torsion, by −27.7°. In the quadruple mutant of HsBR, the C_6_–C_7_ bond is further twisted to −45.1°, and thus a larger spectral shift was predicted (Supplementary Fig. 2).

The QM/MM free energy calculation showed that the free energy of the twisted 6*-s-cis* conformation is lower by −1.1 kcal mol^−1^ than the planar 6*-s-trans* one in the C1C2 mutant, indicating that the twisted 6*-s-cis* conformation is more stable. A small contribution of the chromophore–protein interaction (0.9 kcal mol^−1^) to the free energy difference indicated that the binding pocket was successfully designed to accommodate the twisted 6*-s-cis* chromophore without a significant increase in the steric conflict, despite the large change in its molecular shape.

### Absorption spectra and functions of C1C2 and AR3

**Figure 3:**
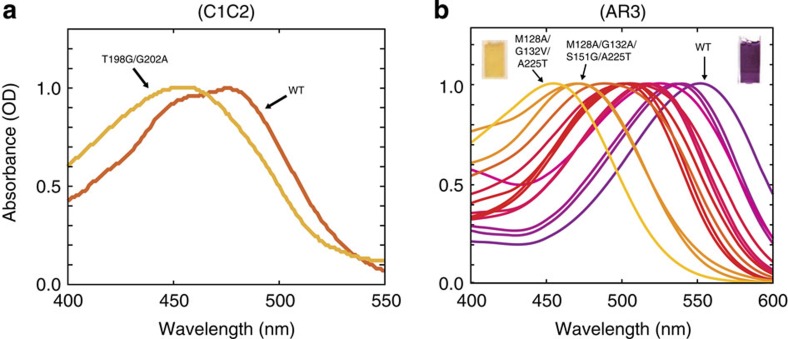
Absorption spectra of wild-type and colour variants of C1C2 and AR3. (**a**) Absorption of spectra of C1C2 and its mutant. (**b**) Absorption spectra of AR3 and its mutants. The solutions of C1C2 contained 150 mM NaCl, 5% glycerol, 0.05% DDM, 0.01% CHS and 50 mM HEPES-NaOH (pH 7.0), and those of AR3 contained 1 M NaCl, 0.05% DDM and 50 mM Tris-HCl (pH 7.0). Absorption maxima of AR3 are summarized in Supplementary Table 4.

To verify the design principle, we measured the absorption spectra and ion transport activities of the designed mutant proteins. First, we expressed and purified the mutants, and measured their absorption spectra. The T198G/G202A mutant of C1C2 (C1C2GA) showed a blue-shifted absorption spectrum (*λ*_max_=455 nm) as compared with that of wild-type C1C2 (C1C2WT; *λ*_max_=476 nm; Fig. 3a), which was well predicted by the torsion at the C_6_–C_7_ bond (Supplementary Table 2 and Supplementary Fig. 2).

Notably, the mutant spectrum lacked the spectral shoulder that appears at ∼447 nm in the C1C2WT spectrum. The spectral shoulder is suggested to represent a vibrational fine structure that is also observed in the absorption spectrum of phoborhodopsin (also known as sensory rhodopsin II, SRII), a microbial rhodopsin functioning as a phototaxis receptor, and experimentally examined by Takahashi *et al*.^43^. The disappearance of the vibrational fine structure is explained well by the rotation of the β-ionone ring.

As suggested by Takahashi *et al*.^43^, the possible causes for the lack of fine structure are a wider distribution of the Franck–Condon vibrational modes in the excited state and/or a ground-state conformational heterogeneity inducing a larger electrostatic perturbation. Although there is no evidence that the distortion around the C_6_–C_7_ bond in the 6*-s-cis* conformation increases the ground-state conformational heterogeneity, the Franck–Condon vibrational distribution in the excited state is expected to be wider in the distorted conformation, since the rotation around the C_6_–C_7_ bond at the distorted angle changes the excitation energy more steeply than that at the planar angle, as shown in Supplementary Fig. 2.

Another possible source for the enhancement of the vibrational fine structure is the contribution of the second excited (S2) state, which lies close to the optically allowed first excited (S1) one^38^,^43^. In this case, the disappearance of the shoulder in the mutant is also well explained by the rotation of the β-ionone ring, which separates the energy levels of the S1 and S2 states (Supplementary Fig. 2), and thus is expected to weaken the absorption intensity of the S2 state by intensity borrowing from the S1 state, through vibronic coupling.

Alternatively, another possible origin of the spectral shoulder in the C1C2WT spectrum is the contribution of the twisted 6*-s-cis* conformation of the retinal chromophore, which may co-exist with the planar 6*-s-trans* conformation in C1C2WT, since the wavelength of the spectral shoulder coincides with the absorption maxima of the C1C2GA mutant. However, from a structural point of view, the X-ray crystallographic structure of C1C2WT^24^ clearly shows that the 6*-s-trans* conformation is precisely recognized by the surrounding protein side chains (Fig. 1), and the 6*-s-cis* conformation in the binding pocket of C1C2WT would give rise to steric clash between the methyl group at the 16th position of the retinal chromophore and the side chain of Thr198. In fact, the present mutation of T198G in the C1C2GA mutant was designed to avoid the steric clash, by reducing the molecular volume of the side chain of Thr198. Other microbial rhodopsins with available structures also possess relatively bulky side chains at the corresponding positions, and were thus found to bind the chromophore in the 6*-s-trans* conformation as well (see Supplementary Fig. 1). C1C2WT is therefore suggested to exclusively bind the 6*-s-trans* conformation, as observed in the other microbial rhodopsins, and thus the spectral shoulder at 450 nm is unlikely to be due to a mixture of the 6*-s-cis* and 6*-s-trans* conformations.

Decomposition of the absorption spectra of C1C2WT and C1C2GA into the spectral components also showed that the spectral width of the component corresponding to the shoulder at 450 nm in the spectrum of C1C2WT is significantly narrower, and is approximately half of that of C1C2GA (Supplementary Fig. 3 and Supplementary Table 3), thus disfavouring the possibility of the mixture of the 6*-s-cis* and 6*-s-trans* conformations in C1C2WT as well.

The absorption maximum of purified AR3, *λ*_max_=552 nm, was also drastically shifted to 454 nm by mutations at only three positions, M128A/G132V/A225T (Fig. 3b). The spectral shift by ∼100 nm of the mutant, which is still functional as a photosensitive ion transporter (see below), is the largest ever produced by the molecular engineering of rhodopsins so far, and is twice as large as the largest one previously obtained for a triple mutant of AR3, M128A/S151A/A225T (*λ*_max_=500 nm)^16^. The blue shift of the absorption spectrum can also be controlled by point mutations at the designed positions. The M128A/G132A/S151G/A225T and M128A/S151A/A225T mutants slightly reduced the shifts to *λ*_max_=471 and 491 nm, respectively (Supplementary Table 4), and single and double mutants at those positions covered the entire spectral range from 450–550 nm (Fig. 3b).

The remarkably large shifts are attained by the non-additive synergy of the multiple mutations (Supplementary Table 4), which clearly indicates the involvement of the atomistically designed conformational changes induced by the coupled mutations in the present study. Especially, the simultaneous mutations at positions 128 and 132 (118 and 122 in HsBR, respectively, depicted in Fig. 2b) gave rise to large synergetic shifts. Moreover, the larger spectral shift of the M128A/G132A/S151G/A225T mutant of AR3 than the shift of the T198G/G202A mutant of C1C2 was predicted by the computational models, in which the C_6_–C_7_ bond of the chromophore in the former is significantly more twisted, as described above (Supplementary Table 2 and Supplementary Fig. 2).

**Figure 4:**
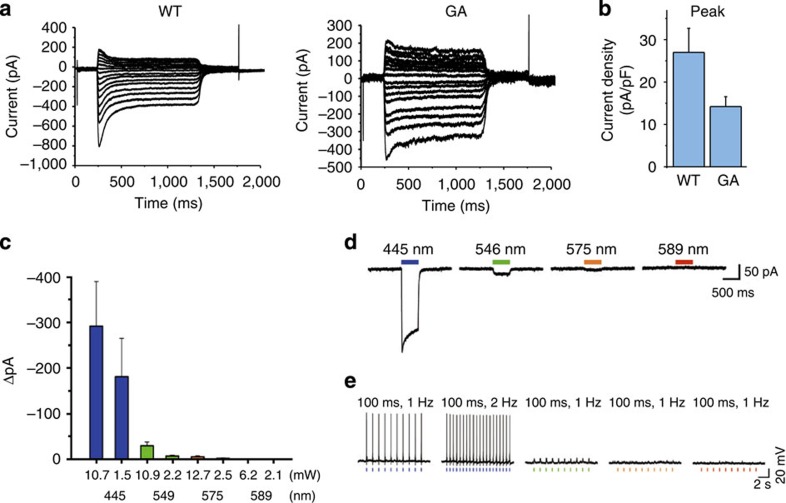
Channel properties of the C1C2GA mutant in HEK cells and mouse neurons. (**a**) Photocurrents of C1C2WT and its GA mutant. Photocurrents in C1C2-expressing HEK293 cells were measured at 16 different holding potentials. (**b**) The peak amplitudes of the photocurrents, normalized by the cell's input capacitance. Values are means and s.e.m. of 8–10 experiments. (**c**,**d**) The peak amplitudes (**c**) and photocurrents (**d**) of the C1C2GA mutant in mouse neurons. (**e**) Current-clamp mode recording of C1C2GA, using light pulses at different wavelengths (100 ms, 1–2 Hz).

Next we examined the photosensitive ion transport activities of the mutants. The C1C2GA mutant expressed in HEK293 cells showed a slightly decreased, but still large photocurrent, as compared with C1C2WT (Fig. 4a,b and Supplementary Fig. 4), and exhibited a blue-shifted action spectrum consistent with its absorption spectrum (Supplementary Fig. 5). C1C2GA, fused to enhanced green fluorescent protein (EGFP), was well expressed in mouse brain neurons (Supplementary Fig. 6), and showed a significantly larger photocurrent (Fig. 4c,d) with light at a shorter wavelength (445 nm) which, in contrast, gives rise to a smaller photocurrent for C1C2WT (Supplementary Fig. 7). Indeed, the optical activation of C1C2GA at 445 nm was sufficient to evoke the neural spikes ensuring the proper function in neurons (Fig. 4e). The photocurrents of the M128A/G132V/A225T mutant of AR3 as well as the wild type were also examined, by measuring the light-induced pH changes of the proteins expressed in *E. coli* (Supplementary Fig. 8a). For the mutant, we found a directionally inverted photocurrent, presumably due to structural changes of the retinal chromophore such as the rotation and/or a position shift of the β-ionone ring induced by the mutations (to be reported and discussed further elsewhere). The action spectra of the magnitude of pH change clearly showed that, similar to the absorption spectra, the activity of the pH change of the mutant is largely enhanced in the shorter wavelength region around 450 nm and is significantly reduced around 550 nm, where the wild-type exhibits the maximal activity (Supplementary Fig. 8b).

### Crystal structure of the C1C2GA mutant

**Figure 5:**
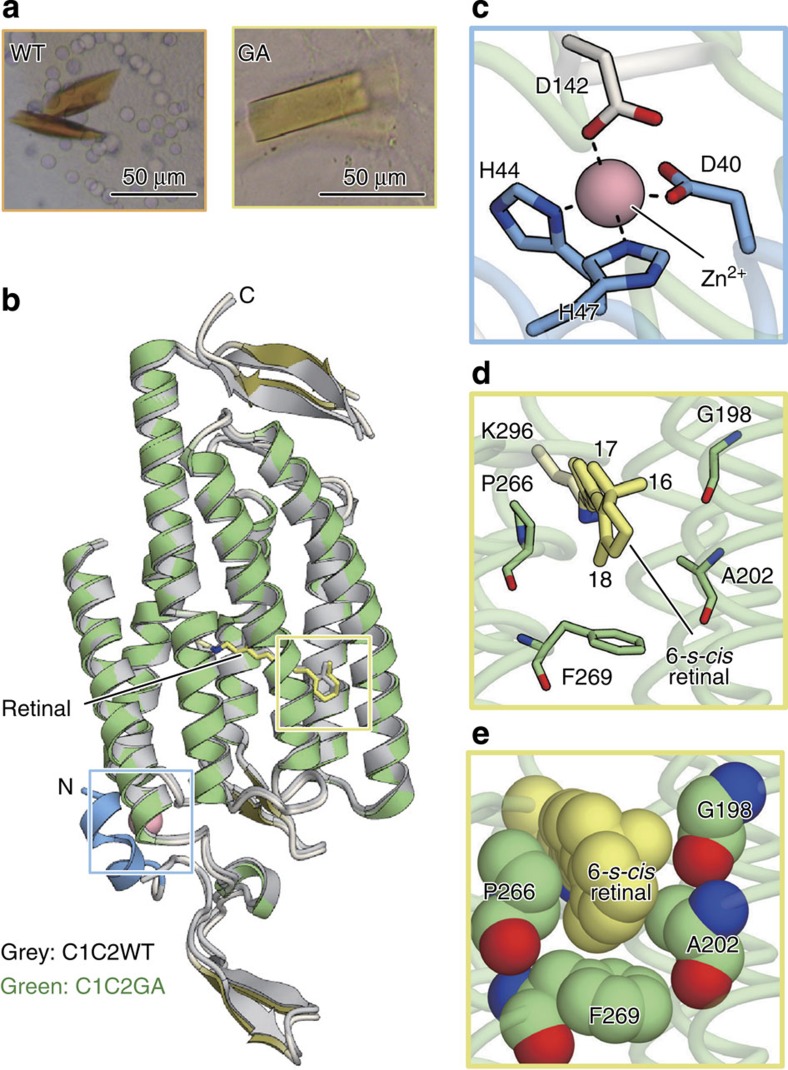
X-ray crystallography of the C1C2GA mutant. (**a**) Photographs of C1C2WT (left) and C1C2GA mutant (right). (**b**) Side view of the superimposed structures of C1C2WT (grey) and C1C2GA (green). The regions around the putative Zn^2+^-binding site and the β-ionone ring of the retinal molecule are highlighted in the blue and yellow rectangles, respectively. The N-terminal region only seen in C1C2GA is coloured blue. The putative Zn^2+^, depicted by a cpk model, is coloured pink, and retinal molecules, depicted by stick models, are coloured grey (C1C2WT) and yellow (C1C2GA), respectively. (**c**) Magnified view of the blue highlighted region in **b**. Pink spheres and black dashed lines represent Zn^2+^ and hydrogen bonds, respectively. (**d**,**e**) Magnified views of the yellow highlighted region in **b**, in which the retinal molecules and surrounding residues are shown as stick models (**d**) and cpk models (**e**).

The atomistically designed structural model that created the blue-shifted spectrum was verified by solving the crystal structure of the C1C2GA mutant. We expressed, purified and crystallized C1C2GA as previously described^24^, and obtained yellow crystals in the lipidic cubic phase under neutral pH conditions (pH 7.0; Fig. 5a,b). The structure was determined by the molecular replacement method, using the coordinates of C1C2WT (PDB ID: 3UG9)^24^, and refined to 2.5 Å resolution (Table 1). As expected, the overall structure of C1C2GA at pH 7.0 is almost identical to that of C1C2WT at pH 6.0 (root mean squared deviation of 0.66 Å over all C_α_ atoms; Fig. 5b), except for the protonation states of titratable groups, the amino-terminal (N-terminal) segment, and the retinal-binding pocket.

Since the pH of the crystallization conditions was different, the protonation states of several titratable residues were altered. We found that the pH differences in the crystallization conditions caused changes in the protonation states of Glu122, Asp40, His44 and/or His47. As a consequence, Glu122, located on the extracellular side of TM2, lost the hydrogen bond with His173 (Supplementary Fig. 9). Since the side chain of Glu122 is completely exposed to the bulk solvent, and the approximate pKa of Glu122 calculated by PROPKA is 5.4, Glu122 may be deprotonated under the neutral pH conditions. On the basis of the crystal structure of C1C2WT at pH 6.0, a previous study suggested that the hydrogen bond between Glu122 and His173 contributes to the formation of the intracellular channel gate 3. Thus, we also assumed that Glu122 is deprotonated and exposed to the bulk solvent, at least in the ground state under physiological conditions, and its contribution to the formation of the channel gate is smaller than previously expected.

The new feature of a binding site of an ion putatively assigned to an endogenous Zn^2+^ appeared at the N-terminal segment in the crystal structure under the neutral pH conditions (Fig. 5c and Supplementary Fig. 10). The electron density map of C1C2GA clearly showed the N-terminal 10 residues (PDYVFHRAHE), which were disordered in the previous C1C2WT structure (Supplementary Fig. 10). These N-terminal residues form a short helix, which is located near the extracellular side of transmembrane helix 2 and extracellular loop 1 (Fig. 5b). Notably, Asp40, His44 and His47 on the N-terminal helix coordinate a cation with Asp142 on ECL1 (Fig. 5c and Supplementary Fig. 10). Owing to the strong electron density and the tetrahedral coordination by the His and Asp residues, this cation is most likely an endogenous Zn^2+^ ion. As far as we know, this is the first evidence to suggest that a putative Zn^2+^ ion is bound to the extracellular side of ChR, and future studies will reveal the physiological role of this ion.

At the binding pocket around the β-ionone ring, significant changes in the electron density due to the mutations were detected. In contrast to C1C2WT, the electron density of C1C2GA clearly indicated the rotation of the β-ionone ring (Supplementary Fig. 11). The space where the C_17_ methyl group resides in C1C2WT becomes occupied by the methyl group of the alanine residue introduced by the mutation, and instead the C_17_ methyl group occupies the pocket formed by the threonine to glycine mutation. The C_5_=C_6_–C_7_=C_8_ dihedral angle changes from 177.7° to −31.4° (counterclockwise, positive), and consequently the retinal configuration changes from 6-*s-trans* to 6-*s-cis*. The atomic resolution structure is in good agreement with the calculated model (Supplementary Fig. 12), and it strongly supports the present design principle that the simultaneous double mutations at positions 198 and 202 in C1C2 (118 and 122 in HsBR, respectively) induce the rotation of the β-ionone ring, which leads to the significant blue-shift of the absorption spectrum.

### Applicability of the design principle

**Figure 6:**
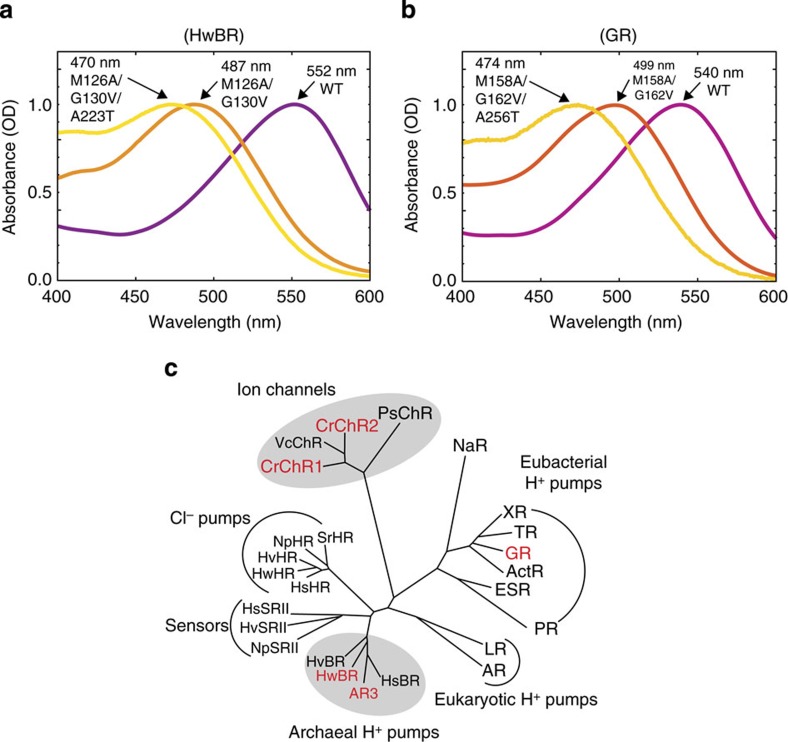
Absorption spectra and phylogenetic tree of HwBR and GR. (**a**,**b**) Absorption spectra of wild-type and colour variants of HwBR (**a**) and GR (**b**). The spectra obtained in the solutions containing 1 M NaCl and 0.05% DDM, with pHs adjusted to 7.0 with 50 mM Tris-HCl. (**c**) Phylogenetic tree of microbial opsins.

The molecular modelling described above suggested that the design principle that guided us to produce the blue-shifted colour variants of C1C2 and AR3 is also applicable to other microbial rhodopsins as well. Therefore, we next measured the absorption spectra of purified mutants of other proton pump microbial rhodopsins, archaeal bacteriorhodopsin from *Haloquadratum walsbyi* (HwBR) and eubacterial rhodopsin from *Gloeobacter violaceus* (GR), engineered based on the same design principle (Fig. 6a,b and Supplementary Table 5). The mutants of HwBR, an orthologue of AR3, showed a large blue shift similar to those of AR3. Furthermore, a phylogenetically distant eubacterial GR from the archaeal rhodopsins (Fig. 6c) also produced similar large blue shifts by the mutations. The similarity of the spectral shifts, together with the blue shifts obtained for the mutant ChR C1C2, seen above, indicates the universality of the molecular mechanism underlying the observed spectral shifts, and consequently the high applicability of the rational approach across species separated by long evolutionary distances (Fig. 6c).

## Discussion

In the present study, colour variants with large spectral shifts were created by altering the conformation of the chromophore, which is a novel and different strategy from that of changing the electrostatic environment surrounding the chromophore, employed in previous studies of retinal-binding proteins^18^,^23^,^44^,^45^. Extensive mutations are not appropriate for producing a large spectral shift of the membrane proteins used as optogenetics tools. The β-ionone ring of the chromophore resides in the very hydrophobic membrane environment, and the introduction of highly polar groups to change the electrostatic environment or poorly packed groups in this region can easily damage the protein stability. Any changes in the electrostatic environment around the Schiff base group of the chromophore, which is situated in the middle of an ion channel, are also limited for preserving the ion transport functionalities, which require precise electrostatic tuning of the ion channels in the hydrophobic membrane environment. In fact, although a directed evolution experiment recently showed that a few combinatory mutations in GR generate blue shifts of absorption spectra by maximally 80 nm, the mutations that created shifts of more than 50 nm were found to abolish the proton pump activity^46^. The high spatial precision of the atomistic design in the present study minimized unnecessary modifications that could lead to loss of ion transport function. Therefore, it was possible to design the synergetic combinatory mutations in the highly packed hydrophobic region around the β-ionone ring, to give rise to the well-controlled conformational changes that remarkably alter the absorption spectra yet maintain the photosensitive ion transport activities. The use of modifications in the region far from the ion channel also leaves room for further engineering to change other properties, such as channel characteristics and protein stability.

The present atomistic design driven by the physicochemical principle successfully identified the universality of molecular recognition for ligand binding, which can be exploited to extensively create blue-shifted colour variants of analogous microbial rhodopsins (Fig. 6). Furthermore, the present design sheds light on the underlying molecular mechanism of the significantly blue-shifted action spectra of the recently isolated ChRs from *Platymonas subcordiformis* and *Tetraselmis cordiformis* (PsChR and TcChR, *λ*_max_=430–40 nm)^14^,^17^. We aligned the sequences of several ChRs, including C1C2WT, C1C2GA, PsChR and TcChR, and found that among all known ChRs, only C1C2GA, PsChR and TcChR simultaneously have glycine residues at position 198 and alanine residues at position 202 (Supplementary Fig. 13). This finding strongly suggests that the blue-shifted absorption spectra of PsChR and TcChR originated from the rotated β-ionone ring of the retinal chromophore, as in C1C2GA. It is noteworthy that no structures of microbial rhodopsins from natural sources that bind the retinal chromophore in the 6*-s-cis* conformation have been found (see Supplementary Fig. 1). The hypothesis of the 6*-s-cis* conformation of the retinal chromophores in PsChR and TcChR may therefore provide novel insight into the architectures of the naturally evolved microbial rhodopsins. The evolutionary view also supports our physicochemical design principle to create the blue-shifted optogenetics tools.

Previously, a triple mutant, M128A/S151A/A225T, of AR3 with a blue shift by ∼50 nm (*λ*_max_=500 nm) was heuristically found, and the shift was tentatively interpreted to be attributed to rotation of the β-ionone ring based on QM/MM modelling^16^. However, much more drastic blue shifts of up to 100 nm with a threefold larger synergetic effect (Supplementary Table 4) were induced by the simultaneous double mutations at positions 128 and 132 (198 and 202 in C1C2), which were absent in the previous mutant and were first introduced in the present study. The observation of the significantly larger shifts indicates that the simultaneous double mutations at positions 128 and 132 are required for the controlled rotation of the β-ionone ring, which was verified by the QM/MM free energy calculation and the X-ray crystallography in the present study. However, the moderate blue shift without the simultaneous double mutations in the previous study is not due to the rotation of the β-ionone ring, but to uncontrolled structural changes.

In optogenetics applications, the colour variants developed in the present study can be employed for simultaneous neural activation and activity imaging^47^,^48^,^49^,^50^ in addition to dual activation^11^,^12^,^13^,^14^,^15^,^16^,^17^. The absorption spectra blue-shifted to ∼450 nm with the steep decay in the red edges significantly widens the no-absorbance region that can be utilized for the activation of red fluorescent proteins for imaging of calcium ions, and thus are expected to improve and augment the simultaneous manipulation/imaging techniques.

The mechanistic generality underlying the design of the blue-shifted mutants established in the present study permits the application of the same design principle for the development of colour variants of other microbial rhodopsins. Although the applicability of equivalent mutants of various microbial rhodopsins as optogenetics tools is not trivial, since the mutations can affect other properties *in vivo* such as expression level, protein stability and photocurrent, the present set of mutations represents a promising first step toward the creation of mutants with significant spectral shifts, which can subsequently be subjected to further maturation for the development of colour variants working as optogenetics tools.

The present study demonstrated that molecular conformational design at atomic resolution, achieved by accurate hybrid molecular simulations and high resolution X-ray crystallography, can create novel variants that are unattainable by conventional rational design. The atomistic design will be a powerful approach to engineer various biotechnological protein tools with complex functionalities, which require precise control of the molecular structures by the mutations.

## Methods

### Molecular simulations

HsBR was used as the model system for AR3 and HwBR, as in the previous study^16^. The X-ray crystallographic structure of HsBR by Luecke *et al*.^20^ (PDB ID: 1C3W) was employed for the initial structure. For the C1C2 chimera, the recently solved X-ray crystallographic structure by Kato *et al*.^24^ (PDB ID: 3UG9) was used. A monomer taken from the X-ray structure of each protein was embedded into a POPC bilayer, and then filled with water molecules and counter ions to neutralize the systems. The numbers of POPC molecules were 125 for HsBR and 118 for C1C2, and those of water molecules were 11,160 for HsBR and 19,757 for C1C2. The titration states of Asp95, Asp115 and Glu204 in HsBR, and those of Glu122, Glu129, Glu136 and Asp195 in C1C2 were set to be protonated. These systems were equilibrated by classical molecular dynamics (MD) calculations. The G122V/M126A/S141G/A215T mutant of HsBR and the T198G/G202A mutant of C1C2 with the retinal protonated Schiff base (RPSB) in the 6*-s-*cis form were also modelled from their corresponding intact proteins. The force field parameters were obtained from the CHARMM22/CMAP parameter set^51^,^52^,^53^,^54^,^55^. For RPSB, the previously determined parameters were employed^29^,^38^,^56^,^57^,^58^,^59^,^60^. Long-range electrostatic interactions were treated with the particle mesh Ewald method. All MD simulations were performed with the NAMD programme package^61^.

The equilibrated systems were further refined by the QM/MM RWFE-SCF method^41^, which combines QM/MM calculations and classical MD simulations. Thus, this method is capable of removing possible structural artifacts of the modelled mutations through sufficient thermal relaxation by MD simulations, while maintaining the high accuracy of the structural determination of the electronically complex chromophore with QM treatment (Supplementary Fig. 14). The QM region consists of the side chain of Lys216 covalently binding RPSB with a boundary at C_β_–C_γ_, Arg82 at C_β_–C_γ_, Asp85 at C_α_–C_β_ and Asp212 at C_α_–C_β_, and three water molecules in the binding pocket (Wat401, Wat402 and Wat406) for HsBR. These QM atoms in HsBR were kept fixed in all of the classical MD trajectory calculations (that is, even in equilibration runs) to maintain the hydrogen bond network in this region. RPSB covalently bound to Lys296, with a boundary at C_δ_-C_ɛ_, was chosen as the QM region for C1C2. Hydrogen atoms were employed for the link atoms capping the QM–MM boundaries. The B3LYP functional was used for the QM/MM calculation in the QM/MM RWFE-SCF geometry optimization. We employed the 6–31+G** basis set for the carboxylate anion groups and water molecules in HsBR, 6–31G for the terminal CH_3_ groups involving link atoms, and 6–31G** for the others. In each cycle of sequential samplings by the classical MD simulations in the QM/MM RWFE-SCF free energy geometry optimizations, equilibrium simulations of 3 ns for HsBR and 4 ns for C1C2 were obtained, and the conformational samples from the last 2 ns for both HsBR and C1C2 were employed for the statistical averages. The sufficiently reweighted averages^41^ were confirmed at the last steps of the free energy optimizations. The convergence criterion of the geometry optimization of the free energy surface for HsBR was set to 1.0 × 10^−3^ Hartree/Bohr for the largest component of the gradient of the QM region. For C1C2, the convergence criterion was decreased to 0.5 × 10^−4^ Hartree/Bohr, to improve the accuracy of the free energy calculation (see below). The QM/MM calculations were performed with a modified version of GAMESS^62^.

To evaluate the relative stability of the 6*-s-*conformers, the free energy difference between the 6-*s*-*trans* and 6-*s*-*cis* conformers of the T198G/G202A mutant of C1C2 was calculated, according to the protocol described previously^42^. The optimized structure of the 6-*s*-*trans* T198G/G202A mutant of C1C2 was obtained by the same procedure as for the 6-*s*-*cis* conformer (Supplementary Table 2). The QM–MM interaction component of the free energy difference between these conformers was calculated using the Bennett acceptance ratio method^63^ (BAR). In the free energy calculation, the structural interpolation of the QM region was divided into the rotation around the C_6_–C_7_ bond by 140° and the remaining part. The rotation part was further divided into 15 points (every 10° from 0 to 140°), and the remaining part was also linearly divided into 10 points. The atomic charges of the QM atoms were linearly changed during the C_6_–C_7_ rotation, and were kept fixed during the calculation in the remaining part. An MD calculation for 40–60 ns was performed at each point, and 20,000 configurations from the last 10 ns of the trajectory were used for the free energy difference calculation with BAR. The total free energy difference between conformers was obtained by adding the QM internal energy component of the free energy to the QM–MM interaction component. The calculated free energy difference, −1.1 kcal mol^−1^, comprised the internal contribution of −2.0 kcal mol^−1^ coming solely from the structural difference of the chromophore, and 0.9 kcal mol^−1^ from the interaction between the chromophore and the surrounding proteins. The free energy difference from the vibrational zero-point energy and the vibrational entropy was ignored, because the contribution is expected to be minor and it exhibited poor convergence behaviour.

### Protein expression and purification of C1C2WT and C1C2GA

The C1C2WT and C1C2GA expression plasmids were constructed as previously described^24^. Virus-infected Sf9 insect cells were cultured in Sf900II (Invitrogen) at 27 °C for 24 h, and then the temperature was reduced to 20 °C. Cells were harvested 72 h after infection. The pellets were disrupted by two passages through a microfluidizer at 15,000 p.s.i., and were resuspended in a buffer containing 300 mM NaCl, 50 mM Tris-HCl, pH 8.0, 5% glycerol and 0.1 mM phenylmethylsulfonyl fluoride. The preparation of the crude membranes and the purification of the proteins were performed essentially according to the previously described methods^24^. Briefly, the cell membranes were solubilized by *n*-dodecyl-β-D-maltoside (DDM) with cholesteryl hemisuccinate (CHS) and purified by chromatography on Ni^2+^ affinity and size-exclusion columns. Peak fractions were collected, concentrated to ∼10 mg ml^−1^ and stored at −80 °C until use.

### Crystallization of C1C2GA

C1C2 was mixed with monoolein (Sigma) in a 2:3 protein to lipid ratio (w/w). Aliquots (100 nl) of the protein-LCP mixture were spotted on a 96-well sandwich plate and overlaid by 1 μl of precipitant solution by the crystallization robot, mosquito LCP (TTP LabTech). Crystals were obtained in 28% (w/v) PEG500DME, 100 mM HEPES-NaOH, pH 7.0, 220 mM Li_2_SO_4_ and 10 mM ATP. All crystals were incubated for 2–3 weeks in the dark. They were harvested using micromounts (MiTeGen), and were flash-cooled in liquid nitrogen without any additional cryoprotectant.

### C1C2 electrophysiology in HEK cells

HEK293 cells were cultured in DMEM, supplemented with 10% fetal bovine serum, on poly-lysine-coated glass-bottom culture dishes (Matsunami). The cells were transfected with 1 μg of the expression plasmid for GFP-tagged C1C2WT or GFP-tagged C1C2GA, using Fugene6 (Promega). After 30 h, the cells were placed under an inverted microscope (IX-71, Olympus), in a bath solution containing 140 mM NaCl, 1 mM CaCl_2_, 2 mM MgCl_2_, 10 mM HEPES and 5 mM glucose (pH 7.4 with NaOH). A borosilicate patch pipette (Harvard Apparatus) was filled with a pipette solution, containing 140 mM KCl, 5 mM EGTA, 2 mM MgCl_2_ and 10 mM HEPES (pH 7.2 with KOH).

Photocurrents were measured in the whole-cell configuration. The cells were held at a membrane potential of −80 mV and depolarized by 10 mV voltage steps up to +70 mV. The light-induced currents were activated 200 ms after the depolarization step, by exposure to 442 nm or 470 nm light for 1,000 ms, elicited with an argon lamp coupled to a Polychrome V monochromator (Till Photonics). Photocurrents were measured with an Axopatch 200B amplifier (Axon CNS, Molecular Devices) connected to an A/D converter, Digidata 1440 (Axon), controlled by the pClamp10.4 software (Axon). Absorption spectra of C1C2WT and the mutant were determined by measuring the photocurrent at −80 mV. Light illuminations were shifted from 340 to 580 nm at 6-nm intervals. Photocurrent intensities were corrected at each wavelength by the power density measured with thermopile (SSC, Japan), and then normalized to the maximum photocurrent. The maximum absorption was estimated at the graph peak. All data were analysed by the pClampfit10.4 (Axon CNS) and Origin (Light Stone) software.

### C1C2 electrophysiology in neurons

Seven-week-old C57BL/6J mice were injected with AAV-CMV-C1C2-2A-mCherry, and killed at 10 weeks of age. All AAV vectors were produced using the AAV Helper-Free System (Agilent Technologies Inc., Santa Clara, CA, USA), and purified basically according to the methods previously reported^64^. In brief, HEK293 cells were transfected with a pAAV vector plasmid that included a gene of interest, pHelper, and pAAV-RC (serotype DJ; purchased from Cell Biolabs Inc., San Diego, CA, USA), using the standard calcium phosphate method. Three days later, the transfected cells were collected and suspended in artificial cerebrospinal fluid (124 mM NaCl, 3 mM KCl, 26 mM NaHCO_3_, 2 mM CaCl_2_ 1 mM MgSO_4_, 1.25 mM KH_2_PO_4_, 10 mM D-glucose). After four freeze–thaw cycles, the cell lysate was treated with benzonase nuclease (Merck, Darmstadt, Germany) at 45 °C for 15 min, and centrifuged twice at 16,000*g* for 10 min. The supernatant was used as the virus-containing solution. To measure the titre of the purified virus dissolved in artificial cerebrospinal fluid, quantitative PCR was performed. The virus was stored at −80 °C in aliquots until use.

Surgeries for AAV injections were conducted under pentobarbital anaesthesia (50 mg kg^−1^, intraperitoneal), using a stereotaxic instrument (David Kopf Instruments, Tujunga, CA, USA). Seven-week-old mice were injected stereotaxically into the LHA with recombinant AAV-CMV-C1C2-2A-mCherry (600 nl per injection, 2 × 1011 copies ml^−1^) with a glass micropipette and an air pressure injector system (Pneumatic PicoPump; World Precision Instruments Inc., Sarasota, FL, USA). Injection sites were as follows: bregma 1.4 mm, lateral ±0.7 mm, ventral −5.0 mm, for AAV-CMV-C1C2-2A-mCherry. Three weeks after the AAV injection, the mice were killed to perform electrophysiological experiments.

Brains were quickly isolated in ice-cold cutting solution, consisting of 280 mM sucrose, 2 mM KCl, 10 mM HEPES, 0.5 mM CaCl_2_, 10 mM MgCl_2_, 10 mM glucose, pH 7.4 with NaOH, bubbled with 100% O_2_, and immediately cut coronally into 350 μm-thick slices with a microtome (VTA-1200S, Leica Microsystems, Wetzlar, Germany). Slices containing the LHA were transferred to an incubation chamber filled with a physiological solution, containing 135 mM NaCl, 5 mM KCl, 1 mM CaCl_2_, 1 mM MgCl_2_, 10 mM HEPES, 10 mM glucose, pH 7.4 with NaOH, bubbled with 100% O_2_. The brain slices were then transferred to a recording chamber (RC-27L, Warner Instruments, Hamden, CT, USA) on a fluorescence microscope stage (BX51WI; Olympus, Tokyo, Japan). The fluorescence microscope was equipped with an infrared camera (C2741-79; Hamamatsu Photonics, Hamamatsu, Japan) for infrared differential interference contrast imaging and a CCD camera (IK-TU51CU, Olympus, Tokyo, Japan). Neurons with mCherry fluorescence were subjected to electrophysiological recordings. Recordings were performed with an Axopatch 200B amplifier (Molecular Devices LLC, Sunnyvale, CA, USA), using a borosilicate pipette (GC150-10; Harvard Apparatus, Holliston, MA, USA) prepared by a micropipette puller (P-1000; Sutter Instruments, Novato, CA, USA), filled with an intracellular solution (4–10 MΩ) consisting of 138 mM K-gluconate, 8 mM NaCl, 10 mM HEPES, 0.2 mM EGTA-Na_3_, 2 mM MgATP and 0.5 mM Na_2_GTP, pH 7.3 with KOH. During recordings, cells were superfused with the extracellular solution at a rate of 1.6 ml min^−1^, using a peristaltic pump (Dynamax; Rainin Instrument LLC, Oakland, CA, USA). For optogenetic stimulation, blue light (475±17.5 nm, 2.5 mW) was generated by a SPECTRA light engine (Lumencor). The output signal was low-pass filtered at 5 kHz and digitized at 10 kHz. Data were recorded on a computer through a Digidata 1322A A/D converter, using the pClamp software (version 10; Molecular Devices). Traces were processed for presentation using the Origin 9 software (Light Stone, Tokyo, Japan) and the Canvas14 software (ACD Systems, Victoria, Canada).

### Protein expression and purification of proton pump microbial rhodopsins

The AR3, HwBR and GR expression plasmids were constructed as previously described^16^. The mutant genes were constructed by PCR, using a QuikChange Site-Directed Mutagenesis Kit (Stratagene, La Jolla, CA, USA) as described previously^65^. All constructed plasmids were analysed using an automated sequencer (ABI 3100) to confirm the expected nucleotide sequences. The *Escherichia coli* DH5α strain was used as the host for DNA manipulation.

For protein expression, transformed *E. coli* BL21(DE3) cells harbouring the plasmid were initially grown at 30 °C in 100 ml LB medium, supplemented with ampicillin (final concentration 50 μg ml^−1^), and were directly inoculated into 2 l LB medium containing ampicillin. The cells were grown overnight on a rotary shaker (MIR-220R, Sanyo Electric Co., Ltd., Osaka, Japan) at 18 °C, in medium containing 0.1% L-arabinose for AR3 and HwBR or 0.5 mM isopropyl-β-D-thiogalactoside for GR and 5 μM all-trans retinal. The cells were harvested by centrifugation at 4 °C, resuspended in buffer (50 mM MES, pH 6.5) containing 1 M NaCl and disrupted by sonication or passage through a French press. The preparation of crude membranes and the purification of proteins were performed essentially by the previously described method^66^. In brief, the cell membranes were solubilized by *n*-dodecyl-β-D-maltoside (DDM, Dojindo Lab, Kumamoto, Japan) and purified by chromatography on an Ni^2+^ affinity column (GE Healthcare UK, Amersham Place, England). When necessary, the samples were further purified by anion exchange chromatography, as described previously^66^. The purified sample was concentrated and buffer exchanged to buffer A (1 M NaCl, 50 mM Tris-Cl and 0.05% DDM, pH 7.0), using an Amicon Ultra filter (Millipore, Bedford, MA, USA).

The proton transport activity was measured by monitoring pH changes, using a glass electrode. The *E. coli* cells expressing AR3 were harvested by centrifugation (4,800*g*, 3 min), and then were washed three times and resuspended in the solvent for the measurement (100 mM NaCl). A 7.5-ml portion of the cell suspension was kept in darkness, and then illuminated with the output of a 100 W Xe arc lamp (LM103, Asahi Spectra, Japan) through a long-pass glass filter for 5 min at a specific wavelength region. The power of the light was controlled by ND filters (0.0625, 0.125, 0.25, 0.5, 1, 2.5 and 5 W) to measure the power dependence of the pumping activities. For the measurement of the action spectra, interference filters (FWHM=10 nm) were used to obtain the light at specific wavelengths, by filtering the output of the same Xe arc lamp. The photon flux of the light was measured by a calibrated multi-channel detector (IRRAD-C2000+, Ocean Optics, USA), and the initial slopes at the specific wavelengths were measured to obtain the action spectrum.

## Additional information

**Accession codes:** Protein Data Bank (PDB). The data for C1C2GA have been deposited under the accession codes 4YZI.

**How to cite this article:** Kato, H. E. *et al*. Atomistic design of microbial opsin-based blue-shifted optogenetics tools. *Nat. Commun.* 6:7177 doi: 10.1038/ncomms8177 (2015).

## Supplementary Material

Supplementary Information Supplementary Figures 1-14, Supplementary Tables 1-5

## Figures and Tables

**Table 1:** Data collection and refinement statistics.

	**C1C2 GA**
Data collection	
Space group	*C*222_1_
Cell dimensions	
*a*, *b*, *c* (Å)	59.6, 142.4, 92.2
Resolution (Å)*	50.0–2.50 (2.56–2.50)
*R*_sym_ or *R*_merge_	14.8 (175.4)
*I*/*σI*	11.8 (1.42)
Completeness (%)	99.9 (100.0)
Redundancy	9.8 (10.0)
CC(1/2)†	99.9 (62.1)
	
Refinement	
Resolution (Å)	37.1–2.50 (2.69–2.50)
No. of reflections	13941
*R*_work_/*R*_free_	22.3/24.1 (30.0/36.6)
No. of atoms	
Protein	2,259
Ligands	78
Water	19
*B*-factors	
Protein	56.3
Ligands	60.1
Water	51.6
r.m.s. deviations	
Bond lengths (Å)	0.003
Bond angles (°)	0.726

r.m.s., root mean squared.

^*^Highest-resolution shell is shown in parentheses.

^†^CC1/2 values were calculated using the programme XDS.
