# Supplementary material for: Atomistic design of microbial opsin-based blue-shifted optogenetics tools
Source: Nat Commun. 2015 May 15;6:7177. doi: 10.1038/ncomms8177 (PMC4479019; doi:10.1038/ncomms8177)
Supplement: Supplementary Information — Supplementary Figures 1-14, Supplementary Tables 1-5 [file ncomms8177-s1.pdf]

Supporting Information for

# Atomistic Design of Microbial Opsin-based Blue-Shifted Optogenetics Tools

Authors: Hideaki E. Kato[1†*], Motoshi Kamiya[2*], Seiya Sugo[2], Jumpei Ito[3], Reiya Taniguchi[1], Ayaka Orito[3], Kunio Hirata[4], Ayumu Inutsuka[5], Akihiro Yamanaka[5], Andrés D. Maturana[3], Ryuichiro Ishitani[1], Yuki Sudo[6], Shigehiko Hayashi[2**], and Osamu Nureki[1**]

Affiliations: [1]*Department of Biological Sciences, Graduate School of Science, The University of Tokyo, 2-11-16 Yayoi, Bunkyo-ku, Tokyo 113-0032, Japan;* [2]*Department of Chemistry, Graduate School of Science, Kyoto University, Kyoto 606-8502, Japan;* [3]*Department of Bioengineering Sciences, Graduate School of Bioagricultural Sciences, Nagoya University, Furo-cho, Chikusa-ku, Nagoya 464-8601, Japan;* [4]*RIKEN SPring-8 Center, Hyogo 679-5148, Japan;* [5]*Department of Neuroscience II, Research Institute of Environmental Medicine, Nagoya University, Nagoya 464-8601, Japan;* [6]*Division of Pharmaceutical Sciences, Graduate School of Medicine, Dentistry, and Pharmaceutical Sciences, Okayama University, 1-1-1 Tsushima-naka, Kita-ku, Okayama 700-8530, Japan.*

*† Present address: Department of Molecular and Cellular Physiology, Stanford University School of*

*Medicine, Stanford, California 94305, USA.*

**Corresponding author. E-mail: hayashig@kuchem.kyoto-u.ac.jp (S.H.); nureki@bs.s.u-tokyo.ac.jp (O.N.)*

Figure Captions for Supplementary figures

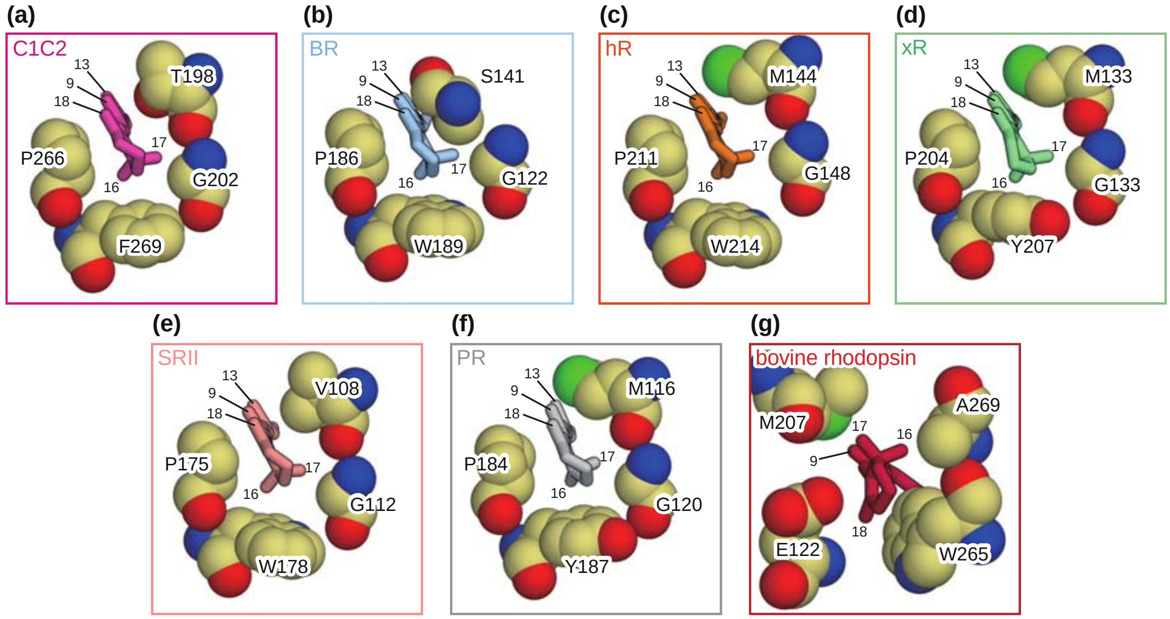


**Supplementary Figure 1**: Comparison of the retinal binding pockets. The retinal binding pockets of C1C2WT (a), BR (b), halorhodopsin (hR) (c), xanthorhodopsin (xR) (d), SRII (e), PR (f), and bovine rhodopsin (g). Retinal molecules are depicted by stick models, and the surrounding residues are shown as cpk models.

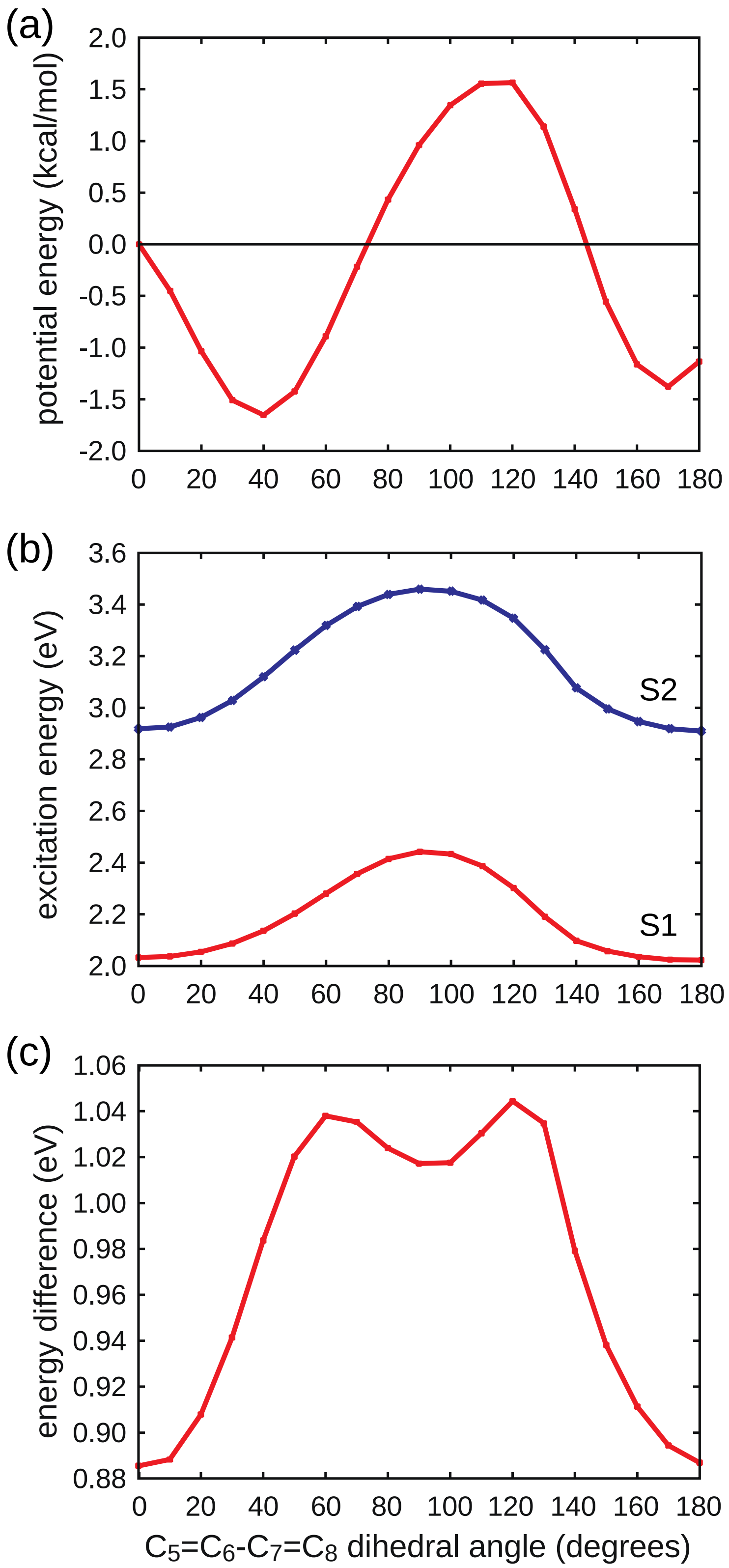

(a)
potential energy (kcal/mol)
2.0
1.5
1.0
0.5
0.0
-0.5
-1.0
-1.5
-2.0
0 20 40 60 80 100 120 140 160 180
(b)
excitation energy (eV)
3.6
3.4
3.2
3.0
2.8
2.6
2.4
2.2
2.0
S2
S1
0 20 40 60 80 100 120 140 160 180
(c)
energy difference (eV)
1.06
1.04
1.02
1.00
0.98
0.96
0.94
0.92
0.90
0.88
0 20 40 60 80 100 120 140 160 180
C5=C6-C7=C8 dihedral angle (degrees)

**Supplementary Figure 2**: Potential energy and excitation energies of RPSB with torsion around the $C_6$-$C_7$ bond in the gas phase. The ground ($S_0$) state geometries of the isolated RPSB moiety, which is the QM region of the QM/MM system of C1C2, along the torsional coordinate were optimized at the M06-2X/6-31G** level of theory. (a) Potential energy curve of the ground state (M06-2X/6-31G**). (b) Changes of the $S_1$ and $S_2$ excitation energies of RPSB. The excitation energies were calculated with the second order extended multi-configuration quasi-degenerate perturbed theory (XMCQDPT2) level of theory with a three-state averaged density, where all the valence π-orbitals and π-electrons of RPSB were involved in the active space and electrons, respectively. Basis functions of 6-31G** were employed for the XMCQDPT2 calculations. (c) Change in the $S_1$-$S_2$ energy difference.

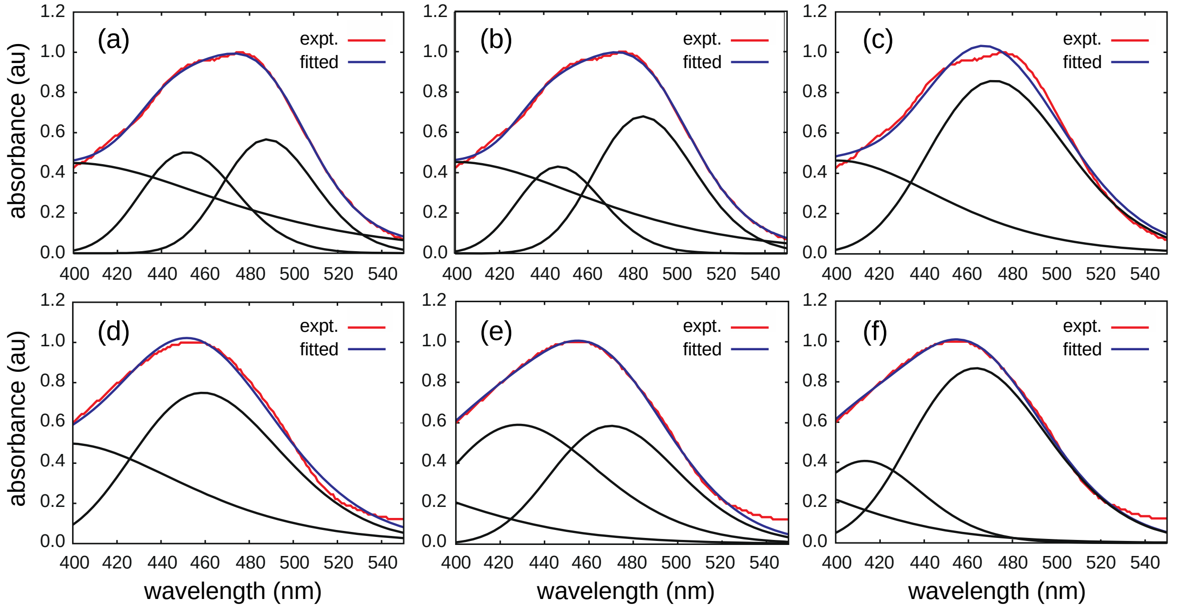


**Supplementary Figure 3:** Decomposition of absorption spectra of C1C2WT and C1C2GA into spectral components expressed by Gaussian functions. Fitted parameters are listed in Supplementary Table 3. Red and blue curves indicate experimental absorption spectra (expt.) and fitted ones (fitted), respectively. Black curves are Gaussian components determined by the fitting. (a) C1C2WT fitted with three Gaussian functions, (b) C1C2WT fitted with three Gaussian functions assuming the same widths for the main and shoulder peaks, (c) C1C2WT fitted with two Gaussian functions, (d) C1C2GA fitted with two Gaussian functions, (e) C1C2GA fitted with three Gaussian functions, and (f) C1C2GA fitted with three Gaussian functions assuming the same widths for two peaks. The absorption spectrum of C1C2WT fit well with three Gaussian functions (a,b). Two sharp Gaussian functions express the main peak and the shoulder peak, respectively, and one broad one at ~72 kcal/mol (Supplementary Table 3) represents a component of the denatured state. In contrast, the absorption spectrum of C1C2WT obviously cannot be expressed with two Gaussian functions (c), indicating that two components are necessary to represent the fine structures of the main band. The absorption spectrum of C1C2GA is well expressed with two Gaussian functions with a consistent component of the denatured state at 72 kcal/mol (Supplementary Table 3) (d), whereas the spectrum failed to be consistently fitted with three Gaussian functions (e,f); components of the denatured state are found at ~80 kcal/mol and are inconsistent with the other fittings. One component is therefore sufficient to represent the main band of C1C2GA.

(a) WT

●:Peak (442 nm)
○:Stable (442 nm)

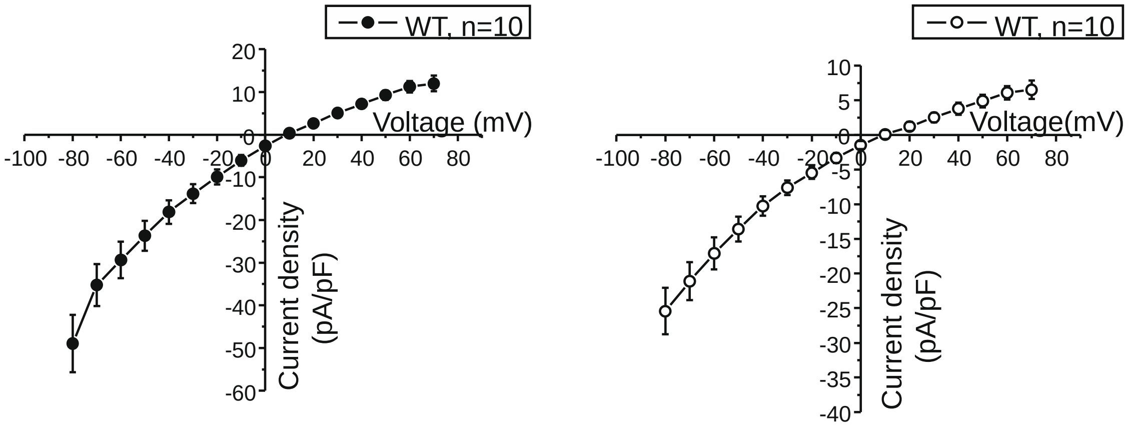


(b) GA

●:Peak (442 nm)
○:Stable (442 nm)

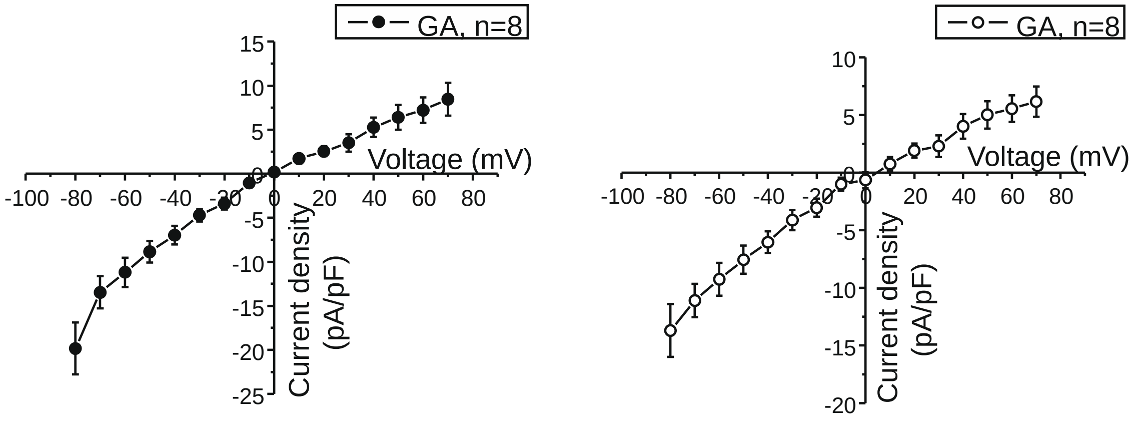


**Supplementary Figure 4**: Current-voltage (I-V) curves of C1C2WT (a) and the C1C2GA mutant (b). The I-V relationships between -80 and + 70mV were determined from the single current amplitude at the indicated potentials. Values are means and SEM of 8-10 experiments.

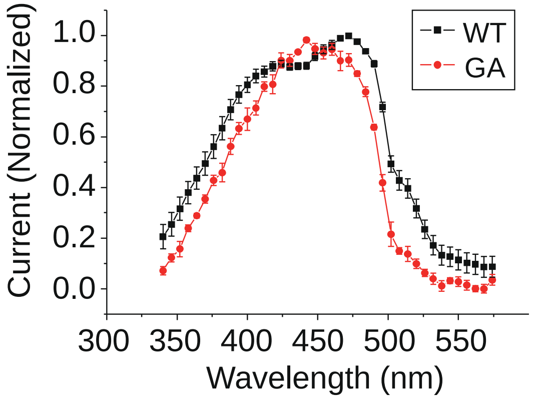


**Supplementary Figure 5**: Action spectra of C1C2WT (black) and C1C2GA (red). Each spectrum is normalized to its own peak for ease of comparison. In the action spectrum of C1C2WT, a second peak at 420 nm appears, in addition to the first peak at 470 nm. Note that the band corresponding to the second peak is absent in the absorption spectrum; the second peak in the action spectrum is located at a significantly shorter wavelength than the sub-band in the absorption maximum (450 nm). The absence of the corresponding peak in the absorption spectrum indicates that the photocurrent at the second peak in the action spectrum does not originate from a state in the resting dark state. From a structural point of view, the binding of the retinal chromophore in the 6-*s*-*cis* conformation in the binding pocket of the wild-type is also unlikely. A likely explanation would be a second conducting state in a branch of the photocycle.

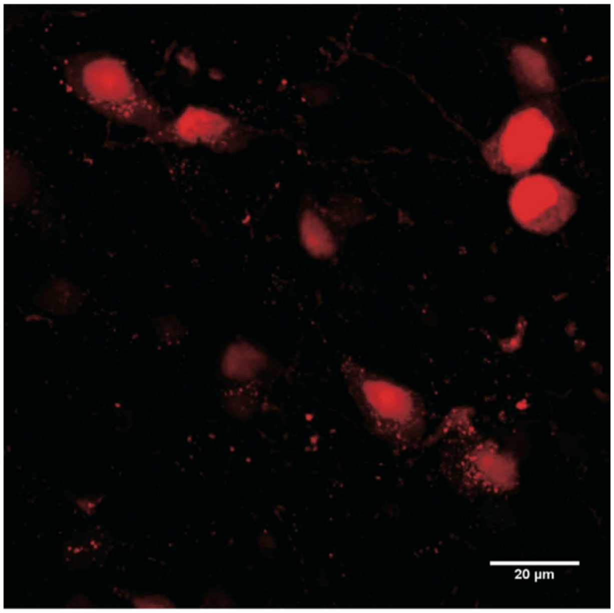


**Supplementary Figure 6**: EGFP-fused C1C2GA expression in neurons. Confocal image of representative mouse neurons expressing C1C2GA. Scale bar represents 20 µm.

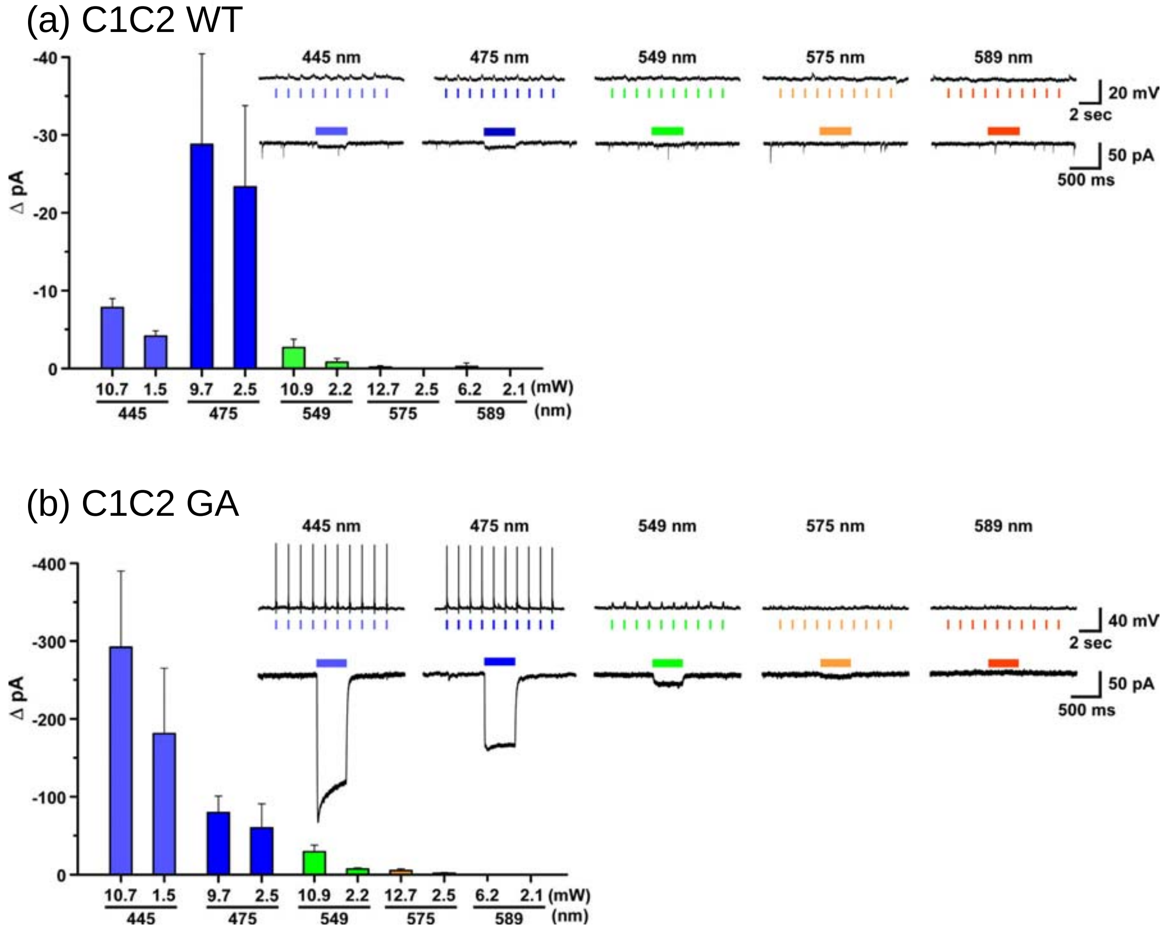


**Supplementary Figure 7**: Channel properties of C1C2WT (a) and C1C2GA (b) in mouse neurons. Peak amplitudes, current-clamp mode recordings, and photocurrents using light pulses at different wavelengths (100 ms, 1-2 Hz) are shown.

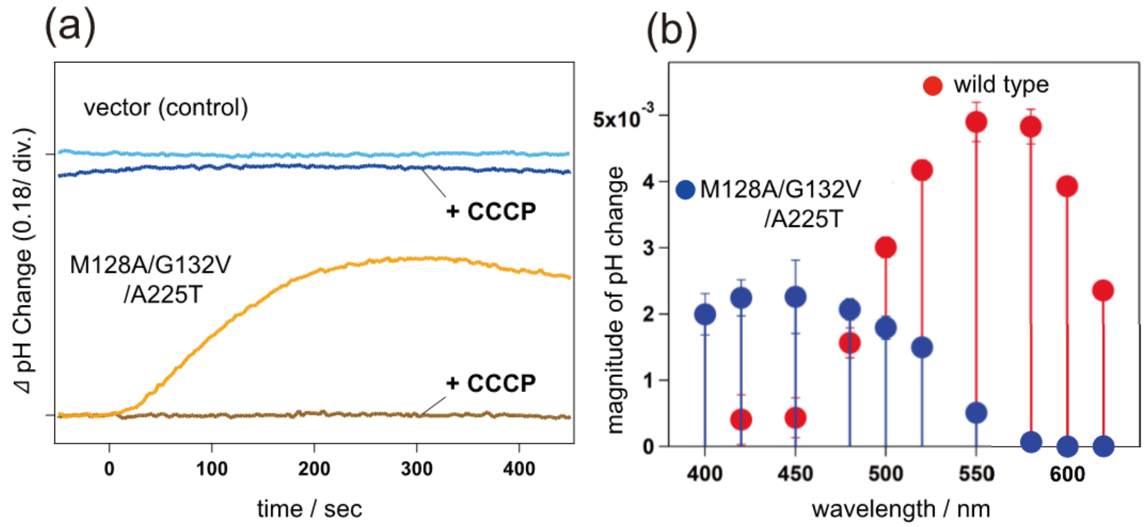


**Supplementary Figure 8**: (a) Light-induced pH change by $H^+$-transport by the M128A/G132V/A225T mutant of AR3. The suspension of *E. coli* carrying only the vector (pBAD24) did not show any pH change upon light illumination. The pH change induced by the M128A/G132V/A225T mutation was cancelled by the addition of a protonophore (CCCP). The light illumination ($\lambda > 430$ nm) began at $t = 0$ s and ceased at $t = 150$ s. (b) Action spectra for the proton pumping activity in transformants containing the wild type (red circles) or the M128A/G132V/A225T mutant (blue circles) of AR3, which were estimated from the initial slopes of the pH change. The error bars indicate the standard deviations of three identical experiments.

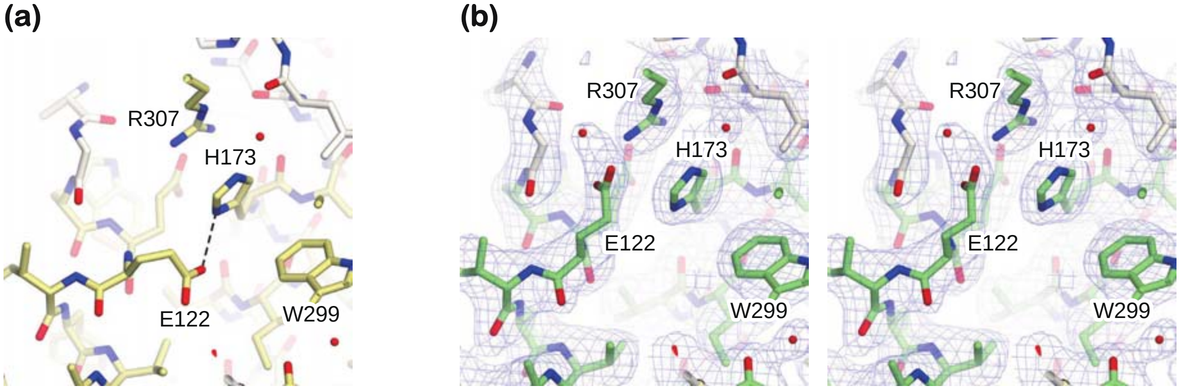


**Supplementary Figure 9**: Structural comparison around the intracellular channel gate between C1C2WT and C1C2GA. (a) The C1C2WT structure around Glu122. (b) Stereo view of the C1C2GA structure in the same region. The 2Fo-Fc map (blue mesh, contoured at 1.0 σ) is shown. Water molecules are shown as red spheres, and the hydrogen bond is represented by the black dashed line.

**(a)**

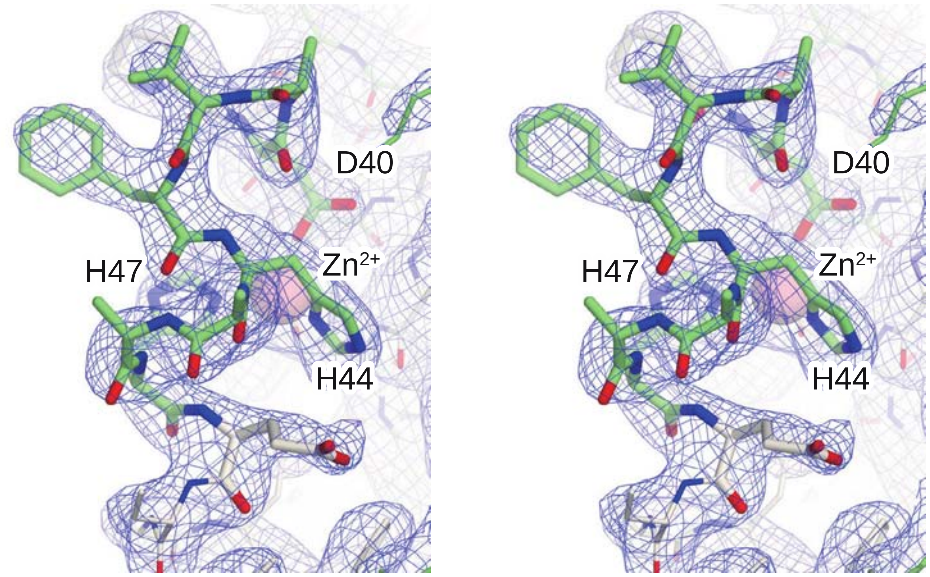


**(b)**

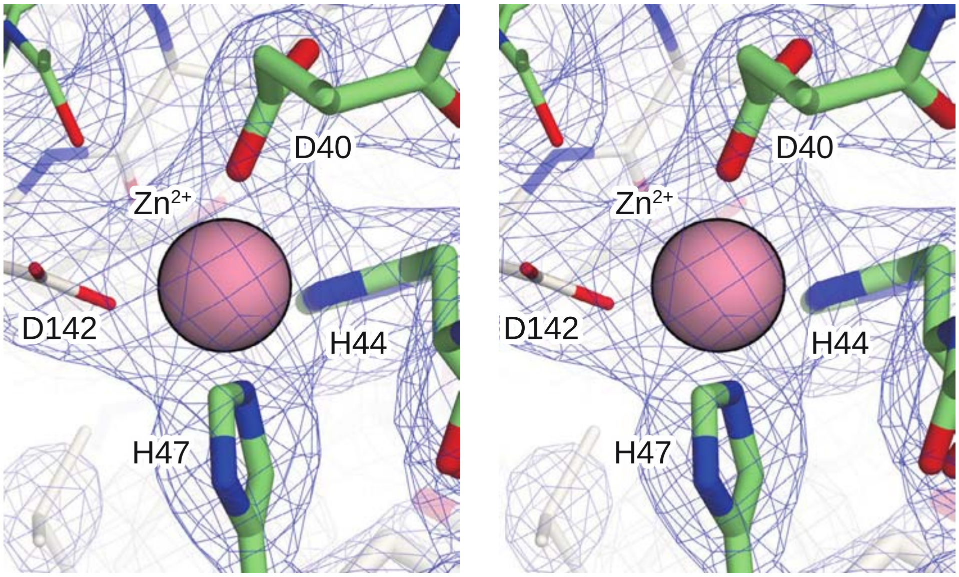


**Supplementary Figure 10**: Electron densities around the N-terminal region and the putative $Zn^{2+}$ binding site. Stereo views of the structures and densities in the overall N-terminal region (a) and the $Zn^{2+}$binding site (b). $Zn^{2+}$ is shown as a cpk model, and the N-terminal 10 residues (PDAVFHRAHE)

are represented as thicker stick models. The $2F_o$-$F_c$ maps (blue mesh, contoured at 1.0 σ) are shown.

**(a)**

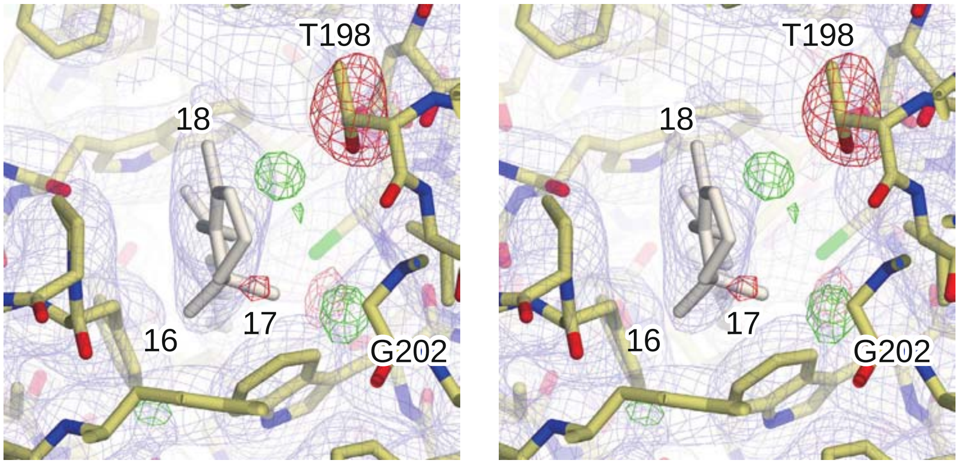


**(b)**

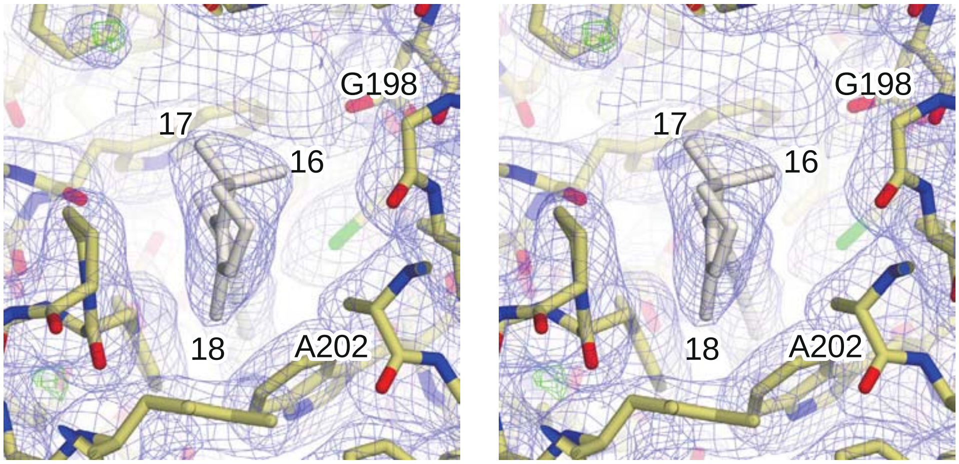


**Supplementary Figure 11**: Electron density maps around the β-ionone ring in the C1C2GA mutant. (a) Stereo views of the structures and densities around the β-ionone ring, just after the molecular replacement using the C1C2WT structure (PDB ID: 3UG9). The $2F_o$-$F_c$ map (blue mesh, contoured at 1.0 σ) and the $F_o$-$F_c$ map (green and red meshes, contoured at 3.0 and -3.0 σ, respectively) are shown. The negative peak at $C_{17}$ and the positive peak between $C_{18}$ and T198 in the $F_o$-$F_c$ map clearly indicate that the β-ionone ring of this mutant is rotated, as compared to that of C1C2 WT. (b) Stereo views of the structures and densities around the β-ionone ring after the structural refinement. The $2F_o$-$F_c$ map (blue

mesh, contoured at 1.0 σ) and the $F_o$-$F_c$ map (green and red meshes, contoured at 3.0 and -3.0 σ, respectively) are shown. This $F_o$-$F_c$ map without any strong negative or positive peak around the β-ionone ring demonstrates that the β-ionone ring of the retinal molecule in C1C2GA mutant is significantly rotated, and the retinal configuration changes from 6-*s*-*trans* to 6-*s*-*cis*.

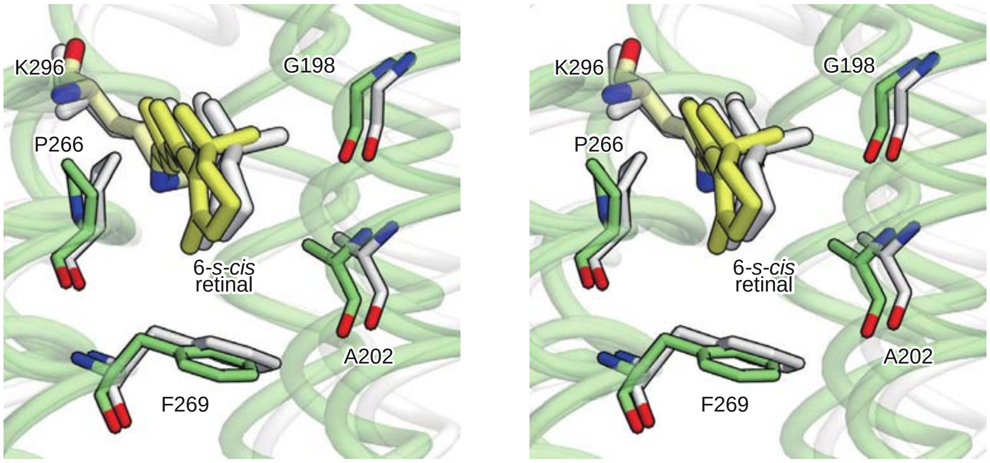


**Supplementary Figure 12**: Structural comparison of the retinal and its binding pocket between the X-ray structure (green) and the calculated model (grey). Retinal molecules are colored yellow (X-ray) and grey (calculated model).

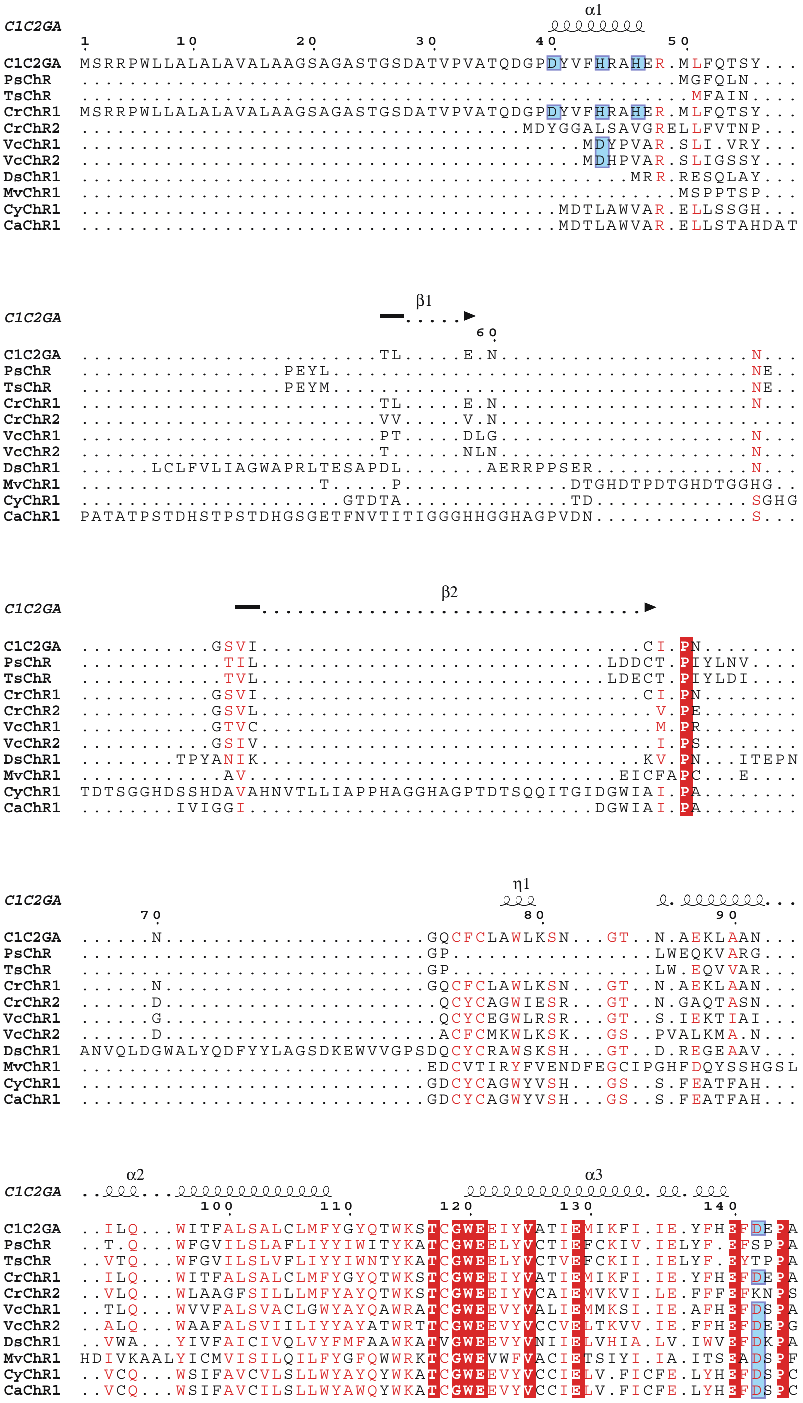
C1C2GA
α1
1 10 20 30 40 50
C1C2GA MSRRPWLLALALAVALAAGSAGASTGSDATVPVATQDGPDYVFHRAHER.MLFQTSY...
PsChR ..............................................MGFQLN....
TsChR ..............................................MFAIN....
CrChR1 MSRRPWLLALALAVALAAGSAGASTGSDATVPVATQDGPDYVFHRAHER.MLFQTSY...
CrChR2 ...................................MDYGGALSAVGRELLFVTNP...
VcChR1 ........................................MDYPVAR.SLI.VRY...
VcChR2 ........................................MDHPVAR.SLIGSSY...
DsChR1 ............................................MRR.RESQLAY...
MvChR1 ..............................................MSPPTSP...
CyChR1 .......................................MDTLAWVAR.ELLSSGH...
CaChR1 .......................................MDTLAWVAR.ELLSTAHDAT
C1C2GA
β1
60
C1C2GA .........................TL.....E.N....................N...
PsChR .................PEYL....................................NE..
TsChR .................PEYM....................................NE..
CrChR1 .........................TL.....E.N....................N...
CrChR2 .........................VV.....V.N.........................
VcChR1 .........................PT.....DLG....................N...
VcChR2 .........................T......NLN....................N...
DsChR1 ......LCLFVLIAGWAPRLTESAPDL.......AERRPPSER.............N...
MvChR1 ....................T.....P.............DTGHDTPDTGHDTGGHG..
CyChR1 ......................GTDTA..............TD.............SGHG
CaChR1 PATATPSTDHSTPSTDHGSGETFNVTITIGGGHHGGHAGPVDN.............S...
C1C2GA
β2
C1C2GA ...........GSVI................................CI.PN........
PsChR ............TIL.............................LDDCT.PIYLNV....
TsChR ............TVL.............................LDECT.PIYLDI....
CrChR1 ...........GSVI................................CI.PN........
CrChR2 ...........GSVL.................................V.PE........
VcChR1 ...........GTVC.................................M.PR........
VcChR2 ...........GSIV.................................I.PS........
DsChR1 ........TPYANIK...............................KV.PN...ITEPN
MvChR1 ............AV................................EICFAPC...E....
CyChR1 TDTSGGHDSSHDAVAHNVTLLIAPPHAGGHAGPTDTSQQITGIDGWIAI.PA........
CaChR1 ........IVIGGI.............................DGWIAI.PA........
C1C2GA
η1
70 80 90
C1C2GA ......N.....................GQCFCLAWLKSN...GT..N.AEKLAAN...
PsChR ............................GP.................LWEQKVARG...
TsChR ............................GP.................LW.EQVVAR...
CrChR1 ......N.....................GQCFCLAWLKSN...GT..N.AEKLAAN...
CrChR2 ......D......................QCYCAGWIESR...GT..N.GAQTASN...
VcChR1 ......G......................QCYCEGWLRSR...GT..S.IEKTIAI...
VcChR2 ......D......................ACFCMKWLKSK...GS..PVALKMA.N...
DsChR1 ANVQLDGWALYQDFYYLAGSDKEWVVGPSDQCYCRAWSKSH...GT..D.REGEAAV...
MvChR1 ............................EDCVTIRYFVENDFEGCIPGHFDQYSSHGSL
CyChR1 ............................GDCYCAGWYVSH...GS..S.FEATFAH...
CaChR1 ............................GDCYCAGWYVSH...GS..S.FEATFAH...
C1C2GA
α2 α3
100 110 120 130 140
C1C2GA ..ILQ...WITFALSALCLMFYGYQTWKSTCGWEEIYVATIEMIKFI.IE.YFHEFDEPA
PsChR ..T.Q...WFGVILSLAFLIYYIWITYKATCGWEELYVCTIEFCKIV.IELYF.EFSPPA
TsChR ..VTQ...WFGVILSLVFLIYYIWNTYKATCGWEELYVCTVEFCKII.IELYF.EYTPPA
CrChR1 ..ILQ...WITFALSALCLMFYGYQTWKSTCGWEEIYVATIEMIKFI.IE.YFHEFDEPA
CrChR2 ..VLQ...WLAAGFSILLLMFYAYQTWKSTCGWEEIYVCAIEMVKVI.LE.FFFEFKNPS
VcChR1 ..TLQ...WVVFALSVACLGWYAYQAWRATCGWEEVYVALIEMMKSI.IE.AFHEFDSPA
VcChR2 ..ALQ...WAAFALSVIILIYYAYATWRTTCGWEEVYVCCVELTKVV.IE.FFHEFDEPG
DsChR1 ..VWA...YIVFAICIVQLVYFMFAAWKATVGWEEVYVNIIELVHIA.LV.IWVEFDKPA
MvChR1 HDIVKAALYICMVISILQILFYGFQWWRKTCGWEVWFVACIETSIYI.IA.ITSEADSPF
CyChR1 ..VCQ...WSIFAVCVLSLLWYAYQYWKATCGWEEVYVCCIELV.FICFE.LYHEFDSPC
CaChR1 ..VCQ...WSIFAVCILSLLWYAWQYWKATCGWEEVYVCCIELV.FICFE.LYHEFDSPC

```
            β3    β4        α4                                     α5
C1C2GA      →  TT  →
               150          160         170         180         190         200

C1C2GA      VIYSSNGNKTVWLRYAEWLLTCPVILIHLSNLTGLANDYNKRTMGLLVSDIGGIVWATTA
PsChR       MIYQTNGEVTPWLRYAEWLLTCPVILIHLSNITGLNDDYSGRTMSLITSDLGGICMAVTS
TsChR       MIFQTNGQVTPWLRYAEWLLTCPVILIHLSNITGLNDDYSGRTMSLITSDLGGICMAVTA
CrChR1      VIYSSNGNKTVWLRYAEWLLTCPVILIHLSNLTGLANDYNKRTMGLLVSDIGTIVWGTTA
CrChR2      MLYLATGHRVQWLRYAEWLLTCPVILIRLSNLTGLSNDYSRRTMGLLVSDIGTIVWGATS
VcChR1      TLWLSSGNGVVWMRYGEWLLTCPVLLIHLSNLTGLKDDYSKRTMGLLVSDVGCIVWGATS
VcChR2      MLYLANGNRVLWLRYGEWLLTCPVILIHLSNLTGLKDDYNKRTMRLLVSDVGTIVWGATA
DsChR1      MLYLNDGQMVPWLRYSAWLLSCPVILIHLSNLTGLKGDYSKRTMGLLVSDIGTIVFGTSA
MvChR1      TLYLTNGQISPQLRYMEWLMTCPVILIALSNITGMAEEYNKRTMTLLTSDVCCIVLGMMS
CyChR1      SLYLSTSNVVNWLRYSEWLLCCPVILIHLSNVTGLSDDYGRRTMGLLVSDIATIVFGVTA
CaChR1      SLYLSTANIVNWLRYSEWLLCCPVILIHLSNVTGLSDDYGRRTMGLLVSDIATIVFGITA

                                      α6                              α7
C1C2GA
               210         220         230         240         250         260

C1C2GA      ALSK.GYVRVIFFLMG..LCYGIYTFFNAAKVYIEAYHTVPKGRCRQVVTGMAWLFFVSW
PsChR       ALSK.GWLKWLFFVIG..CCYGASTFYHAALIYIESYYTMPHGVCKNMVLAMAAVFFTSW
TsChR       ALSK.GWLKALFFVIG..CGYGASTFYNAACIYIESYYTMPQGICRRLVLWMAGVFFTSW
CrChR1      ALSK.GYVRVIFFLMG..LCYGIYTFFNAAKVYIEAYHTVPKGICRDLVRYLAWLYFCSW
CrChR2      AMAT.GYVKVIFFCLG..LCYGANTFFHAAKAYIEGYHTVPKGRCRQVVTGMAWLFFVSW
VcChR1      AMCT.GWTKILFFLIS..LSYGMYTYFHAAKVYIEAFHTVPKGICRELVRVMAWTFFVAW
VcChR2      AMST.GYIKVIFFLLG..CMYGANTFFHAAKVYIESYHTVPKGLCRQLVRAMAWLFFVSW
DsChR1      ALAPPNHVKVILFIIG..LLYGLFTFFTAAKVYIEAYHTVPKGQCRNLVRAMAWTYFVSW
MvChR1      AASK.PRLKGILYAVG..WAFGAWTYWTALQVYRDAHKAVPKP.LAWYVRAMGYVFFTSW
CyChR1      AMLV.NWPKIIFYLIGFTMC..CYTFFLAAKVLIESFHQVPKGICRHLVKAMAITYFVGW
CaChR1      AMLV.SWPKIIFYLLGFTMC..CYTFYLAAKVLIESFHQVPKGICRHLVKAMAITYYVGW

                                      α8                      α9
C1C2GA
               270         280         290         300         310

C1C2GA      GMFPILFILGPEGFGVLSVYGSTVGHTIIDLMSKNCWGLLGHYLRVLI...........H
PsChR       FMFPGLFLAGPEGTNALSWAGSTIGHTVADLLSKNAWGMIGHFLRLEI...........H
TsChR       FMFPGLFLAGPEGTQALSWAGTTIGHTVADLLSKNAWGMIGHFLRVEI...........H
CrChR1      AMFPVLFLLGPEGFGHINQFNSAIAHAILDLASKNAWSMMGHFLRVKI...........H
CrChR2      GMFPILFILGPEGFGVLSVYGSTVGHTIIDLMSKNCWGLLGHYLRVLI...........H
VcChR1      GMFPVLFLLGTEGFGHISPYGSAIGHSILDLIAKNMWGVLGNYLRVKI...........H
VcChR2      GMFPVLFLLGPEGFGHLSVYGSTIGHTIIDLLSKNCWGLLGHFLRLKI...........H
DsChR1      AMFPILFILGREGFGHITYFGSSIGHFILEIFSKNLWSLLGHGLRYRI...........R
MvChR1      LTFPGWFLLGPEGLEVVTGTVSTLMHACSDLISKNLWGFMDWHLRVLVARHHRKLFKAEE
CyChR1      SFFPLIFLFGQSGFKKISPYADVIASSFGDLISKNAFGMLGHFLRVKI...........H
CaChR1      SFFPLIFLFGQSGFKKISPYADVIASSFGDLISKNMFGLLGHFLRVKI...........H

                            β5            β6
C1C2GA
                  320         330         340

C1C2GA      EHILIHGDI......RK..TTKL.NIGGTEIEVETL.....V....EDEAEAGAV.....
PsChR       KHIIIHGDV......RRPITVN..TL...GREV.TV..SCFVDKEEEDE.DERISTKTYA
TsChR       KHIIIHGDV......RRPVTVKALGR...QVSVNCF...VDKEEEEEDE.RI........
CrChR1      EHILLYGDI......RK..KQKV.NVAGQEMEVETM.....V..HEEDDE..........
CrChR2      EHILIHGDI......RK..TTKL.NIGGTEIEVETL.....V....EDE.AEAGAVP...
VcChR1      EHILLYGDI......RK..KQKI.TIAGQEMEVETL.....V...AEEE.D.........
VcChR2      EHILLYGDI......RK..VQKI.RVAGEELEVETL.....M....TEE.APDTVKKSTA
DsChR1      QHIIIHGNL......TK..KNKI...NIAGDNVEVE.....E..................
MvChR1      EHALKKGQTLEPGMPRS..TSFV.RGLGDDVEIDPSYELYRLKRQNHPEYFL........
CyChR1      EHILKHGDI......RK..TTHL.RIAGEEKEVETF.....V....EEE...........
CaChR1      EHILKHGDI......RK..TTHL.RIAGEEKEVETF.....V....EEEDEDTVKHSTKE

C1C2GA

C1C2GA      ..................................................
PsChR       NRASFMKMRNDMEQRGIQTRKSLEMLAPPPALNDGSIVLAVADPMTLTFF
TsChR       ..................................................
CrChR1      ..................................................
CrChR2      ..................................................
VcChR1      ..................................................
VcChR2      QYANRESFLTMRDKLKEKGFEVRASLDNSGIDAVINHNNNYNNALAN...
DsChR1      ..................................................
MvChR1      ..................................................
CyChR1      ..................................................
CaChR1      LANRGSFIVMRGNMKAQGIDVRASLDMEEDEEGGMGKGKGKGAG......
```

**Supplementary Figure 13**: Sequence alignment. Shown are C1C2GA, ChR from *Platymonas subcordiformis* (PsChR, GenBank ID: AGF84747.1), ChR from *Tetraselmis striata* (TsChR, GenBank ID: AHH02155.1), ChR1 from *Chlamydomonas reinhardtii* (CrChR1, GenBank ID: 15811379), ChR2 from *Chlamydomonas reinhardtii* (CrChR2, GenBank ID: 158280944), ChR1 from *Volvox carteri* (VcChR1, UniProtKB ID: B4Y103), ChR2 from *Volvox carteri* (VcChR2, UniProtKB ID: B4Y105), ChR1 from *Dunaliella salina* (DsChR1, GenBank ID: AEY68833.1), ChR1 from *Mesostigma viride* (MvChR1, GenBank ID: 338176939), ChR1 from *Chlamydomonas yellowstonensis* (CyChR1, GenBank ID: AER58217.1), and ChR1 from *Chlamydomonas augustae,* (CaChR1, GenBank ID: AER58220.1). The C-termini are truncated. Secondary structure elements for C1C2 are shown as coils (α: α-helices, η: $3_{10}$-helices) and arrows (β-strands). Identical and conservatively substituted residues are highlighted in red. The residues contributing to the putative $Zn^{2+}$ binding site are colored cyan. The glycine at position 198 and the alanine at position 202 (in C1C2GA) are colored green.

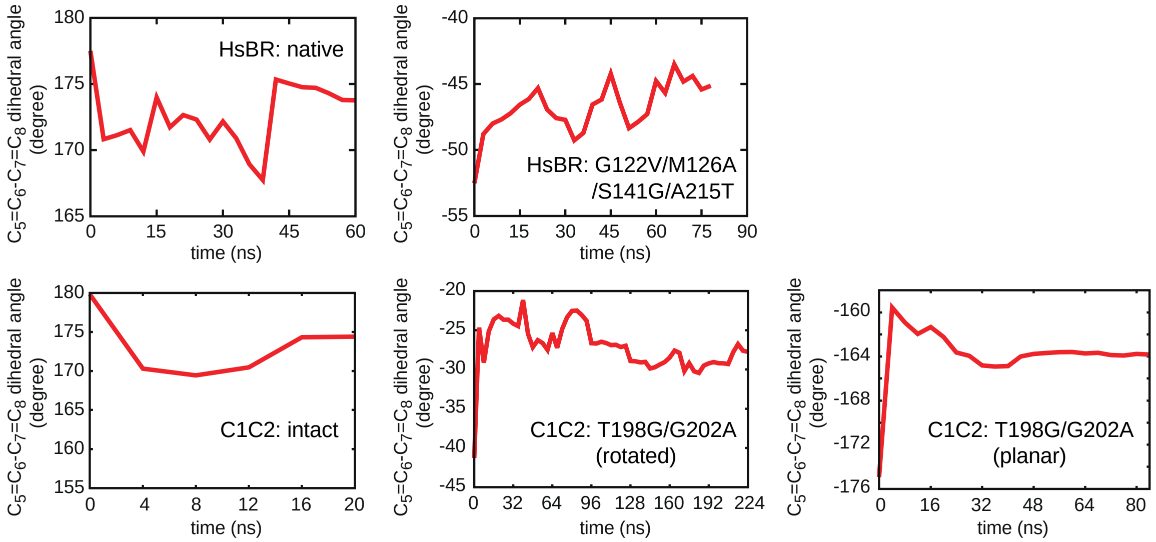


**Supplementary Figure 14**: Evolution of the dihedral angle around the $C_5$-$C_6$ bond in the RWFE free energy geometry optimization. Large changes of the dihedral angles and their convergence in the free energy geometry optimizations with MD trajectories for tens of nano-seconds were observed, indicating that the dihedral angles were well refined from the initial models without becoming trapped at a local minimum, which often occurs in a potential energy geometry optimization.

Supplementary Tables

**Supplementary Table 1**: Dihedral angles of $C_5$=$C_6$-$C_7$=$C_8$ in the structurally known rhodopsins.

| | Dihedral angle of $C_5$-$C_6$-$C_7$-$C_8$ (°) |
|---|---|
| C1C2WT (PDB ID: 3UG9) | 177.7 |
| BR (PDB ID: 1C3W) | 176.3 |
| HR from *H. salinarum* (PDB ID: 1E12) | 168.4 |
| HR from *N. pharaonis* (PDB ID: 3A7K) | 178.0 |
| XR (PDB ID: 3DDL) | -179.3 |
| SRII (PDB ID: 1H68) | 178.0 |
| Blue PR (PDB ID: 4JQ6) | -179.9 |
| AR2 (PDB ID: 3AM6) | 178.7 |
| ASR (PDB ID: 1JGJ) | 177.5 |
| Archaerhodopsin-1 (PDB ID: 1UAZ) | -179.9 |
| Archaerhodopsin-2 (PDB ID: 1VGO) | 178.7 |
| | |
| Bovine rhodopsin (PDB ID: 1F88) | -59.44 |
| C1C2GA (this study) | -31.43 |

**Supplementary Table 2:** Torsional angles of $C_5$=$C_6$-$C_7$=$C_8$ of RPSB in the computational models (degrees).

| C1C2 | | HsBR | |
|---|---|---|---|
| intact | T198G/G202A | native | M118A/G122A/S141G/A215T[b] |
| 174.4 | -27.7 (-163.9[a]) | 173.8 | -45.1 |

[a] 6-*s*-*trans* conformation.

[b] Mutations in HsBR corresponding to the M128A/G132A/S151G/A225T mutations in AR3.

**Supplementary Table 3:** Fitting parameters of decomposition of absorption spectra of C1C2WT and C1C2GA shown in Supplementary Fig. 3[a].

C1C2WT: three Gaussian functions

| $n$ | $a_n$ | $b_n$ / (kcal/mol)$^{-2}$ | $c_n$ / kcal/mol |
|---|---|---|---|
| 1 | 0.567 | 0.0793 | 58.7 |
| 2 | 0.503 | 0.0547 | 63.4 |
| 3 | 0.449 | 0.00512 | 71.5 |

C1C2WT: three Gaussian functions assuming the same widths for the main and shoulder peaks

| $n$ | $a_n$ | $b_n$ / (kcal/mol)$^{-2}$ | $c_n$ / kcal/mol |
|---|---|---|---|
| 1 | 0.680 | 0.0670 | 59.0 |
| 2 | 0.431 | | 64.1 |
| 3 | 0.453 | 0.00582 | 71.5 |

C1C2WT: two Gaussian functions

| $n$ | $a_n$ | $b_n$ / (kcal/mol)$^{-2}$ | $c_n$ / kcal/mol |
|---|---|---|---|
| 1 | 0.859 | 0.0318 | 60.7 |
| 2 | 0.464 | 0.00894 | 71.4 |

C1C2GA: two Gaussian functions

| $n$ | $a_n$ | $b_n$ / (kcal/mol)$^{-2}$ | $c_n$ / kcal/mol |
|---|---|---|---|
| 1 | 0.750 | 0.0247 | 62.4 |
| 2 | 0.498 | 0.00720 | 72.1 |

C1C2GA: three Gaussian functions

| $n$ | $a_n$ | $b_n$ / (kcal/mol)$^{-2}$ | $c_n$ / kcal/mol |
|---|---|---|---|
| 1 | 0.590 | 0.0181 | 66.8 |
| 2 | 0.585 | 0.0365 | 60.9 |
| 3 | 0.324 | 0.00544 | 80.7 |

C1C2GA: three Gaussian functions assuming the same widths for two peaks

| $n$ | $a_n$ | $b_n$ / (kcal/mol)$^{-2}$ | $c_n$ / kcal/mol |
|---|---|---|---|
| 1 | 0.407 | 0.0298 | 69.3 |

| | | | |
|---|---|---|---|
| 2 | 0.867 | | 61.8 |
| 3 | 0.338 | 0.00628 | 80.0 |

[a] The absorption intensity $g(x)$ is fitted by Gaussian functions $g(x) = \sum_n a_n \exp(-b_n (x - c_n)^2)$, where $x$ is the absorption energy in kcal/mol.

**Supplementary Table 4:** Absorption maximum, opsin shift and retinal configuration of wild type (WT) AR3 and various mutants.

| Opsin type | $\lambda_{max}$ [nm] | $\Delta\upsilon$ [$cm^{-1}$] | $\Delta\Delta\upsilon$ [$cm^{-1}$][b] | all-*trans* [%][c] |
|---|---|---|---|---|
| WT | 552[a] | - | - | 53 ± 2[a] |
| M128A | 536[a] | 541[a] | - | 68 ± 1[a] |
| G132V | 526 | 895 | - | 65 ± 3 |
| A225T | 540 | 403 | - | 70 ± 2 |
| | | | | |
| M128A/G132V | 471 | 3115 | 1679 | 75 ± 3 |
| M128A/S151G | 505 | 1686 | ND | 51 ± 3 |
| M128A/A225T | 518 | 1189 | 245 | 51 ± 3 |
| G132V/A225T | 508 | 1569 | 271 | 75 ± 2 |
| | | | | |
| M128A/S151G/A225T | 505 | 1686 | -403 | 41 ± 2 |
| M128A/S151A/A225T | 491 | 2251 | ND | 71 ± 2 |
| M128A/G132V/A225T | 454 | 3910 | 2071 | 65 ± 3 |
| | | | | |
| M128A/G132A/S151G/A225T | 471 | 3115 | ND | 51 ± 3 |
| M128A/G132V/S151G/A225T | 461 | 3576 | -324 | 70 ± 1 |

ND: not determined.

[a] From Sudo et al. (2013) J. Biol. Chem.

[b] Differences between the spectral shift of the mutant and the sum of the shifts of the mutants with fewer replacements, indicating the synergetic effect of the spectral shift.

[c] The molar composition of each retinal isomer was calculated from the areas of the peaks in the HPLC patterns.

**Supplementary Table 5.** Absorption maximum, opsin shift, and retinal configuration of WT and various mutants of HwBR and GR.

| Opsin type | $\lambda_{max}$ [nm] | $\Delta\upsilon$ [cm$^{-1}$] | all-*trans* [%] [b] |
|---|---|---|---|
| (HwBR)-WT | 552[a] | - | 79 ± 2[a] |
| M126A/G130V | 487 | 2418 | 75 ± 3 |
| M126A/G130V/A223T | 472 | 3070 | 70 ± 1 |
| | | | |
| (GR)-WT | 540 | - | 90 ± 3 |
| M158A | 535 | 173 | 72 ± 2 |
| G162V | 504 | 1323 | 93 ± 2 |
| M158A/G162V | 498 | 1562 | 93 ± 3 |
| M158A/G162V/A256T | 474 | 2579 | 64 ± 2 |

[a] From Sudo et al. (2013) J. Biol. Chem.

[b] The molar composition of each retinal isomer was calculated from the areas of the peaks in the HPLC patterns.
